# Stretching the structural envelope of imatinib to reduce β-amyloid production by modulating both β- and γ-secretase cleavages of APP

**DOI:** 10.3389/fchem.2024.1381205

**Published:** 2024-10-08

**Authors:** William J. Netzer, Anjana Sinha, Mondana Ghias, Emily Chang, Katherina Gindinova, Emily Mui, Ji-Seon Seo, Subhash C. Sinha

**Affiliations:** ^1^ Laboratory of Molecular and Cellular Neuroscience, The Rockefeller University, New York, NY, United States; ^2^ Appel Alzheimer’s Disease Research Institute, Feil Family Brain and Mind Research Institute, Weill Cornell Medicine, New York, NY, United States

**Keywords:** imatinib (IMT) or gleevec, DV2-103, gamma secretase, inhibitor, modulator, Aβ, isomer

## Abstract

We previously showed that the anticancer drug imatinib mesylate (IMT, trade name: Gleevec) and a chemically distinct compound, DV2-103 (a kinase-inactive derivative of the potent Abl and Src kinase inhibitor, PD173955) lower Aβ levels at low micromolar concentrations primarily through a lysosome-dependent mechanism that renders APP less susceptible to proteolysis by BACE1 without directly inhibiting BACE1 enzymatic activity, or broadly inhibiting the processing of other BACE1 substrates. Additionally, IMT indirectly inhibits γ-secretase and stimulates autophagy, and thus may decrease Aβ levels through multiple pathways. In two recent studies we demonstrated similar effects on APP metabolism caused by derivatives of IMT and DV2-103. In the present study, we synthesized and tested radically altered IMT isomers (IMTi’s) that possess medium structural similarity to IMT. Independent of structural similarity, these isomers manifest widely differing potencies in altering APP metabolism. These will enable us to choose the most potent isomers for further derivatization.

## 1 Introduction

Neurotoxic β-amyloid peptides (Aβ) are major drivers of Alzheimer’s disease (AD) and are formed by sequential cleavage of the amyloid precursor protein (APP) by β-secretase (BACE1/2) and γ-secretase, respectively. Both β- and γ-secretases can be pharmacologically inhibited to reduce production of Aβ peptides. Indeed, there has been great interest in the development of inhibitors and modulators of the secretases as potential AD therapeutics (Miranda et al., 2021; Kumar et al., 2018; Zhao et al., 2020; Portelius et al., 2010; Hur, 2022; Panza et al., 2009; Golde et al., 2013; Rynearson et al., 2021) but at this time all clinical trials involving secretase inhibitors/modulators have failed. Reasons given have included timing of drug administration (too late in disease course for benefits to occur); non-specific inhibition of secretase substrates other than APP; lack of target engagement; toxicity; and even failure of the Amyloid hypothesis (Kim et al., 2022).

In our previous study, we have shown that the anticancer drug IMT, which is a potent Abl kinase inhibitor (Buchdunger et al., 1996) and PD173955 (Nagar et al., 2002), an Abl/Src kinase inhibitor, reduce Aβ production in cultured N2a695 cells, rat embryonic neurons, and in guinea pig brain *in vivo* by indirectly inhibiting γ-secretase processing of APP, while sparing γ-secretase processing of Notch1 in cellular assays (Netzer et al., 2003). In a recent study we further showed that a kinase inactive derivative of PD173955, DV2-103, as well as IMT, reduced Aβ levels in cells mainly by indirectly inhibiting BACE cleavage of APP (Netzer et al., 2017), adding to our earlier study suggesting that the Aβ-lowering effect of IMT and DV2-103 are not only Abl kinase-independent but also broadly kinase-independent and affect both γ-secretase and BACE processing of APP. IMT and DV2-103 decrease levels of APP-βCTF and sAPPβ, and raise levels of APP-αCTF, as well as a 141 amino acid APP-CTF (C141), and a 9 kDa APP-CTF (all consistent with reduced BACE processing of APP) in N2a695 cells (Netzer et al., 2017). Remarkably, this pattern of APP metabolites induced by IMT and DV2-103, and some of their analogs is observed when N2a695 cells are treated with a general, active-site-directed BACE inhibitor (Netzer et al., 2017; Sun et al., 2019; Sinha et al., 2019). We demonstrated that IMT does not inhibit BACE1 enzymatic activity in two *in vitro* BACE1 assays at concentrations up to 100 μM or inhibit processing of several non-APP BACE substrates in cells (Netzer et al., 2017) and that the inhibitory activities of IMT and DV2-103 require acidified lysosomes. We provided a model suggesting that the effects of these drugs on APP metabolism were a result of their effects on lysosomes, which caused APP to undergo increased trafficking to lysosomes and spend less time in the amyloidogenic pathway where Aβ and its direct precursor, the APP-βCTF, are formed (Netzer et al., 2017).

**FIGURE 1:**
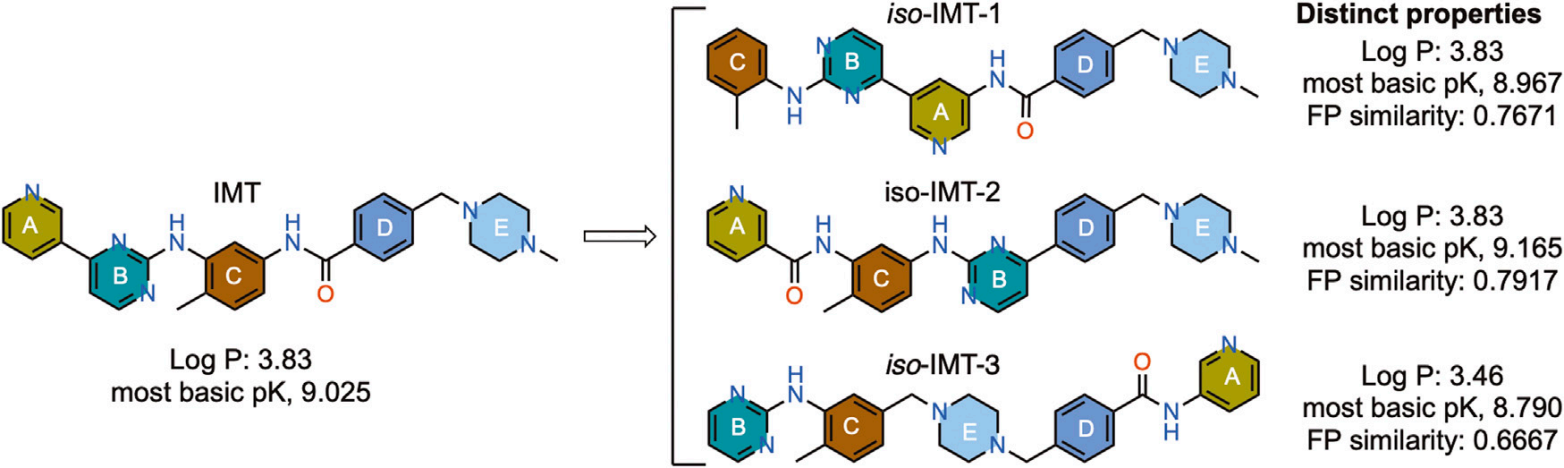
Structure and properties of IMT and the designed IMT isomers. Fingerprint (FP) similarity of IMTi-1 – 3 to parent IMT and their properties, including most basic PK, were calculated *in silico.*

To understand how so many structurally different compounds reduce levels of secreted Aβ in cells and have a characteristic effect on APP metabolite levels, we designed novel IMT isomers, IMTi-1 – 3 (Figure 1A), and tested their effects on APP metabolism by measuring the Aβ levels in cell supernatants and APP metabolites in cell lysates. The design involved a large change in the structure of IMT to greatly alter the pharmacophore structurally but maintain IMT’s physical properties, in particular its property as a weak base, which is necessary for its sequestration in lysosomes through ion trapping (Kazmi et al., 2013; Burger et al., 2015). We further developed and evaluated 28 new analogs of the more potent IMT isomers, IMTi-1 and 2, to gain structure-activity relationship among the new analogs and to the previously described IMT analogs. The results of our studies described in this article support that the pharmacophores of IMT greatly affect APP processing and introduce IMTi-1 and 2 as new pharmacophores to further develop more potent analogs that may function similarly to IMT.

## 2 Materials and methods

All commercial chemicals and solvents were reagent grade and used without further purification. All air-sensitive reactions were performed under argon protection. Column chromatography was performed using 230–400 mesh silica gel. Analytical thin layer chromatography was performed on 250 μM silica gel F_254_ plates. Preparative thin layer chromatography was performed on 1,000 μM silica gel F_254_ plates. All final compounds were purified using HPLC. The identity and purity of each product was determined using MS, HPLC, TLC, and NMR analyses. ^1^H NMR spectra were recorded on either a Bruker 400 or 600 MHz instrument. Chemical shifts are reported in δ values in ppm downfield from TMS as the internal standard. ^1^H data are reported as follows: chemical shift, multiplicity (s = singlet, d = doublet, t = triplet, q = quartet, br = broad, m = multiplet), coupling constant (Hz), integration. Purity of target compounds has been determined to be >95% by LC/MS on a Waters purification system with PDA, MicroMass ZQ and ELSD detector and a reverse phase column (Waters X-Bridge C18, 4.6 × 150 mm, 5 µm) eluted with water/acetonitrile gradients, containing 0.1% TFA. All compounds tested in this study were prepared in house and their structures were confirmed using ^1^H NMR and MS analyses (Spectral data provided for new compounds only). Yields are from a single reaction and not optimized. All final compounds were obtained in >95% purity as judged by LCMS.

N2a695 were cultured in 1:1 OptiMem Reduced Serum Media (Life Technologies): Dulbecco’s Modified Eagle Medium ([+] 4.5 g/L D-glucose [+] L-Glutamine; [−] Sodium pyruvate (Life Technologies) supplemented with 5% fetal bovine serum, 0.4% Penstrep and 0.4% Geneticin and incubated at 37°C in 5% CO_2_. Antibodies were obtained from The Laboratory of Molecular and Cellular Neuroscience at The Rockefeller University. Human Aβ40 and Aβ42 ELISA plates (Life Technologies) and Plus MSD (Mesoscale Discovery) plates for Aβ Peptide (Aβ38, Aβ40 and Aβ42) Panel 1 (6E10) Kit (Catalog number K15200G) were obtained from Thermo Fisher, Life Technologies and Meso Scale Discovery.

### 2.1 Synthesis of IMTi-1 and analogs 1a-r

i)  **Intermediates 5a-b.** To a solution of **4a** (350 mg, 1.3 mmol) and o-toluidine (0.3 mL) in *i*-PrOH (3 mL) was added 1N HCl (1.5 mL), and the mixture was heated at 125°C using Microwave for 2 h. Solvents were removed under reduced pressure, residues treated with aqueous NaHCO_3_ to neutralize, and the resulting mixture extracted with EtOAc. The combined organic layers were washed with brine, dried over anhydrous MgSO_4_, concentrated, and purified by Combi-Flash over Silica gel column using hexanes-EtOAc as eluents to afford intermediate **5a** (300 mg, 68%). ^1^H NMR (600 MHz, CDCl_3_) of **5a**: δ 9.16 (s, 1H), 8.85 (s, 1H), 8.51 (s, 2H), 8.03 (d, *J* = 7.92 Hz, 1H), 7.35–7.30 (m, 2H), 7.14–7.07 (m, 2H), 2.38 (s, 3H); HRMS: *m/z* 341.0391 and 343.0370 [M + H]^+^.
Similarly, intermediate **4b** (270 mg, 1 mmol) was reacted with o-toluidine (0.25 mL) in *i*-PrOH (2 mL) and 1N HCl (1 mL) using the method described for **5a** to afford intermediate **5b** (245 mg, 72%). ^1^H NMR: (600 MHz, CDCl_3_) of **5b**: δ 8.47 (d, *J* = 6.0 Hz, 1H), 8.22 (s, 1H), 8.09 (d, *J* = 6.0 Hz, 1H), 7.96 (d, *J* = 6.0 Hz, 1H), 7.62 (d, *J* = 6.0 Hz, 1H), 7.36 (t, *J* = 6.0 Hz, 1H), 7.29 (t, *J* = 6.0 Hz, 1H), 7.24 (d, *J* = 12 Hz, 1H), 7.11 (d, *J* = 6.0 Hz, 1H), 7.06 (t, *J* = 6.0 Hz, 1H), 6.95 (s, 1H), 2.37 (s, 3H); HRMS: *m/z* 340.0439 and 342.0418, [M + H]^+^.
ii)  **Compound IMTi-1 (General Buchwald coupling method).** A solution of **5a** (38 mg) and **6a** (21 mg) in dioxane was degassed and charged with Pd_2_(dba)_3_ (4 mg), XanthPhos (7 mg), and Cs_2_CO_3_ (55 mg) and heated at 100°C temperature for 16 h. Solvents were removed and worked up using EtOAc and water. Combined organic layers were dried over anhydrous MgSO_4_ and concentrated under reduced pressure. The resulting residues were purified by preparative TLC (Silica gel, 1 mm plate; CH_2_Cl_2_:MeOH:Aq. NH_3_ (90:10:1)) to afford the target product IMTi-1 (35 mg, 63%). ^1^H NMR (400 MHz, CDCl_3_): δ 9.01 (d, *J* = 1.5 Hz, 1H), 8.92 (s, 1H), 8.88 (s, 1H), 8.47 (d, *J* = 5.12 Hz, 1H), 8.05 (d, *J* = 7.96 Hz, 1H), 7.88 (d, *J* = 7.96 Hz, 2H), 7.45 (d, *J* = 8.40 Hz, 1H), 7.28 (t, *J* = 4.28 Hz, 1H), 7.23 (d, *J* = 7.40 Hz, 1H), 7.15 (d, *J* = 5.16 Hz, 1H), 7.07–7.03 (m, 2H), 3.57 (s, 2H), 2.49 (br s, 8H), 2.35 (s, 3H), 2.30 (s, 3H); HRMS: *m/z* 494.2653 [M + H]^+^. Purity (HPLC): >98%.
iii)  **Compound 1a.** Buchwald coupling of **5b** (48 mg, 0.14 mmol) with **6a** (33 mg, 0.14 mmol) in 1,4-Dioxane (3 mL) was performed by heating the mixture at 100°C in the presence of Pd_2_(dba)_3_ (5 mg), XanthPhos (8 mg), and Cs_2_CO_3_ (68 mg) for 16 h. Usual work-up and purification afforded **1a** (33 mg, 48%). ^1^H NMR (400 MHz, CDCl_3_): δ 8.46 (d, *J* = 5.20 Hz, 1H), 8.36 (s, 1H), 8.12 (d, 8.04 Hz, 1H), 8.02 (s, 1H), 7.87–7.82 (m, 4H), 7.52–7.46 (m, 3H), 7.31–7.23 (m, 1H), 7.17 (d, *J* = 5.16 Hz, 1H), 7.06 (t, *J* = 3.6 Hz, 1H), 6.96 (s, 1H), 3.56 (s, 2H), 2.52 (br s, 8H), 2.37 and 2.33 (s, 3H each); HRMS: *m/z* 493.2671 [M + H]^+^.
iv)  **Compounds 1b-Boc and 1b.** Buchwald coupling of **5a** (160 mg, 0.47 mmol) with **6b** (155 mg, 0.49 mmol) in 1,4-Dioxane (10 mL) was performed by heating the mixture at 100°C in the presence of Pd_2_(dba)_3_ (20 mg), XanthPhos (27 mg), and Cs_2_CO_3_ (155 mg) for 16 h. Usual work-up and purification afforded **1b**-**Boc** (100 mg, 35%). MS of **1b-Boc**: *m/z* 579.30.
**Boc-deprotection.** To a solution of **1b-Boc** (100 mg) in EtOAc (3 mL) was added 4 M HCl in dioxane (1 mL) at room temperature (RT) and the mixture stirred overnight (16 h). Solvents were removed under reduced pressure and worked up using CH_2_Cl_2_ and Aq. NaHCO_3_ solution to afford compound **1b** (79 mg, 95%). HRMS: *m/z* 480.2467 [M + H]^+^.
v)  **Compound 1c.** Intermediate **5a** (75 mg, 0.2 mmol) underwent Buchwald coupling with **6c** (53 mg, 0.24 mmol) in the presence of Pd_2_(dba)_3_ (10 mg), Xanthphos (13 mg), and CS_2_CO_3_ (72 mg) in 1,4-Dioxane (5 mL) overnight at 95°C, as described above for IMTi-1 to give compound **1c** (56 mg, 40%). ^1^H NMR (600 MHz, CDCl_3_): δ 9.04 (s, 1H), 8.94 (s, 1H), 8.86 (d, *J* = 6.0 Hz, 1H), 8.13 (dd, *J* = 12.0, 6.0 Hz, 1H), 7.87 (d, *J* = 6.0 Hz, 2H), 7.32 (t, *J* = 6.0 Hz, 2H), 7.265 (d, *J* = 6.0 Hz, 1H), 7.215 (d, *J* = 6.0 Hz, 1H), 7.08 (t, *J* = 6.0 Hz, 1H), 6.975 (d, *J* = 6.0 Hz, 2H), 3.40 (t, *J* = 6.0 Hz, 4H), 2.62 (t, *J* = 6.0 Hz, 4H), 2.40 and 2.39 (s, 3H each); MS: *m/z* 480.25 [M + H]^+^.
vi)  **Compound 1d.** Intermediate **5b** (145 mg, 0.42 mmol) underwent Buchwald coupling with **6c** (93 mg, 0.42 mmol) in the presence of Pd_2_(dba)_3_ (19 mg), XanthPhos (25 mg), and Cs_2_CO_3_ (206 mg) in 1,4-Dioxane (10 mL) overnight at 100°C as described above and worked up and purified to give compound **1d** (110 mg, 55%). ^1^H NMR (600 MHz, CDCl_3_) of **1k**: δ 8.49 (d, *J* = 6.0 Hz, 1H), 8.36 (s, 1H), 8.17 (dd, *J* = 6.0, 12.0 Hz, 1H), 7.88–7.83 (m, 3H), 7.51 (t, *J* = 6.0 Hz, 1H), 7.33–7.29 (m, 2H), 7.27 (d, *J* = 6.0 Hz), 7.22 (d, *J* = 6.0 Hz, 1H), 7.09 (t, *J* = 6.0 Hz, 1H), 6.99 (d, *J* = 6.0 Hz, 2H), 3.41 (t, *J* = 6.0 Hz, 4H), 2.63 (br t, 4H), 2.41 and 2.40 (s, 3H each); MS: *m/z* 479.25 [M + H]^+^.
vii)  **Compound 1e.** Intermediate **5a** (35 mg, 0.1 mmol) underwent Buchwald coupling with amide **6d** (21 mg, 0.11 mmol) in the presence of Pd_2_(dba)_3_ (4 mg), XanthPhos (6 mg), and Cs_2_CO_3_ (50 mg, 0.15 mmol) at 100°C overnight to give compound **1e** (20 mg, 44%) after usual work-up using EtOAc and Aq. NH_4_Cl solution, and purification. ^1^H NMR (600 MHz, CDCl_3_): δ 9.03, 8.95 and 8.90 (s, 1H each), 8.50 (d, *J* = 6.0 Hz, 1H), 8.09 (d, *J* = 6.0 Hz, 1H), 7.87 (d, *J* = 6.0 Hz, 2H), 7.35 (d, *J* = 6.0 Hz, 2H), 7.32–7.29 (m, 1H), 7.25 (d, *J* = 6.0 Hz, 1H), 7.20 (d, *J* = 6.0 Hz, 1H), 7.08 (d, *J* = 6.0 Hz, 2H), 2.89 (t, *J* = 6.0 Hz, 2H), 2.59 (d, *J* = 6.0 Hz, 2H), 2.37 (s, 3H), 2.33 (s, 6H); HRMS: *m/z* 453.2358 [M + H]^+^.
viii)  **Compound 1f.** Intermediate **5b** (340 mg, 1 mmol) underwent Buchwald coupling with amide **6d** (200 mg, 1 mmol) in the presence of Pd_2_(dba)_3_ (45 mg, 5 mol%), XanthPhos (60 mg, 10 mo%), and Cs_2_CO_3_ (500 mg, 1.5 mmol). Usual work-up after heating at 100°C for 16 h and purification gave compound **1f** (270 mg, 60%). ^1^H NMR (600 MHz, CDCl_3_): δ 8.49 (d, *J* = 6.0 Hz, 1H), 8.38 (s, 1H), 8.15 (d, *J* = 6.0 Hz, 1H), 7.99 (br s, 1H), 7.88–7.83 (m, 4H), 7.68 (d, *J* = 6.0 Hz, 1H), 7.53 (t, *J* = 6.0 Hz, 1H), 7.42–7.38 (m, 2H), 7.33 (d, *J* = 12.0 Hz, 1H), 7.26 (d, *J* = 6.0 Hz, 1H), 7.21 (d, *J* = 6.0 Hz, 1H), 7.08 (t, *J* = 6.0 Hz, 1H), 6.97 (s, 1H), 2.29 (q, *J* = 6.0 Hz, 2H), 2.61 (q, *J* = 6.0 Hz, 2H), 2.40 (s, 3H), 2.38 (s, 6H); HRMS: *m/z* 452.2450 [M + H]^+^. Purity (HPLC): >98%.
ix)  **Compound 1g.** Intermediate **5a** (38 mg, 0.11 mmol) underwent Buchwald coupling with **6e** (22 mg, 0.15 mmol) in the presence of Pd_2_(dba)_3_ (5 mg), XanthPhos (10 mg), and Cs_2_CO_3_ (73 mg mmol) in 1,4-Dioxane (2 mL) by heating overnight at 100°C. Sovents were removed under reduced pressure and the residues purified by preparative TLC to give compound **1** **g** (15 mg, 31%). HRMS: *m/z* 439.2243 [M + H]^+^.
x)  **Compounds 1h-Boc and 1h.** Buchwald coupling of **5a** (60 mg, 0.18 mmol) with **6f** (56 mg, 0.18 mmol) in 1,4-Dioxane (3 mL) was performed by heating the mixture at 100°C in the presence of Pd_2_(dba)_3_ (8 mg), XanthPhos (10 mg), and Cs_2_CO_3_ (86 mg) for 16 h. Usual work-up and purification afforded **1h-Boc** (71 mg, 70%). ^1^H NMR (400 MHz, CDCl_3_) of **1h-Boc**: δ 9.04 (s, 1H), 8.43 (s, 1H), 8.85 (s, 1H), 8.49 (d, *J* = 5.04 Hz, 1H), 8.07 (d, *J* = 8.0 Hz, 1H), 7.89–7.87 (br, 2H), 7.27–7.17 (m, 6H), 7.07–7.03 (m, 2H), 4.58 (br, 2H), 3.16 (br s, 1H), 3.07 (br s, 1H), 2.36 (s, 3H), 1.50 and 1.34 (s, 6H and 3H); 0.97 (s, 9H); MS: *m/z* 581.32 [M + H]^+^.
Compound **1h-Boc** (65 mg, 0.11 mmol) in EtOAc (3 mL) was stirred with 4 M HCl in dioxane (1 mL) at RT for 16 h. Solvents were removed under reduced pressure and worked-up using CH_2_Cl_2_ and Aq. NaHCO_3_ solution to afford compound **1h** (42 mg, 77%). ^1^H NMR (400 MHz, CDCl_3_) of **1h**: δ 9.06 (s, 1H), 8.94 (s, 1H), 8.85 (s, 1H), 8.50 (d, *J* = 5.04 Hz, 1H), 8.10 (d, *J* = 8.0 Hz, 1H), 8.07 (d, *J* = 8.0 Hz, 1H), 8.0 (s, 1H), 7.89 (d, *J* = 7.92 Hz, 2H), 7.52 (d, *J* = 7.80 Hz, 2H), 7.24–7.21 (m, 4H), 7.08 (t, *J* = 4.00 Hz, 1H), 7.00 (s, 1H), 3.92 (s, 2H), 2.38 (s, 2H), 2.37 (s, 3H), 0.95 (s, 9H); HRMS: *m/z* 481.2616 [M + H]^+^.
xi)  **Compounds 1i-Boc and 1i.** Intermediate **5b** (40 mg, 0.12 mmol) was reacted with **6f** (38 mg, 0.12 mmol) under Buchwald coupling conditions using Pd_2_(dba)_3_ (2 mg), XanthPhos (4 mg), and Cs_2_CO_3_ (30 mg) in 1,4-Dioxane (2 mL) overnight as described above at 100°C to give the Boc-protected derivative, **1i-Boc** (45 mg, 66%). MS of **1i-Boc**: *m/z* 580.33 [M + H]^+^.
  Compound **1i-Boc** (40 mg, 0.07 mmol) was deprotected using 4 M HCl in dioxane (1 mL) at RT. Solvents were removed under reduced pressure and worked up using CH_2_Cl_2_ and Aq. NaHCO_3_ solution to afford compound **1f** (30 mg, 90%). ^1^H NMR (400 MHz, CDCl_3_) of **1i**: δ 8.47, 8.36 and 8.14 (s, 1H each), 7.88–7.83 (m, 4H), 7.50 (d, *J* = 7.40 Hz, 4H), 7.24–7.19 (m, 3H), 7.08–7.01 (m, 2H), 3.90 (s, 2H), 3.49 (s, 2H), 2.34 (s, 3H), 0.95 (m, 9H); HRMS: *m/z* 480.2776 [M + H]^+^.
  xii)  **Compounds 1j-Boc and 1j.** Intermediate **5a** (19 mg) was reacted with **6g** (20 mg) under Buchwald coupling conditions using Pd_2_(dba)_3_ (2 mg), XanthPhos (4 mg), and Cs_2_CO_3_ (30 mg) in 1,4-Dioxane (2 mL) overnight at 100°C to give **1j-Boc** (15 mg, 46%). ^1^H NMR (400 MHz, CDCl_3_) of compound **1j-Boc**: δ 9.04 (br s, 1H), 8.97 and 8.88 (s, 1H each), 8.72 (br, 1H), 8.47 (d, *J* = 4.28 Hz, 1H), 8.06 (d, *J* = 7.92 Hz, 4H), 7.94 (m, 2H), 7.44 7.36–7.17 (m, 5H), 7.06 (m, 2H), 4.42 (br s, 2H), 4.08 (m, 1H), 2.36 (s, 3H), 1.70–1.4 (m, 8H), 1.35–1.26 (m, (9H+2H); MS: *m/z* 593.32 [M + H]^+^.
  Compound **1j-Boc** (15 mg, 0.025 mmol) was deprotected using 4 M HCl in dioxane (0.5 mL) at RT. Solvents were removed under reduced pressure and worked up using CH_2_Cl_2_ and Aq. NaHCO_3_ solution to afford compound **1f** (10 mg, 81%). ^1^H NMR (400 MHz, CDCl_3_) of compound **1j**: δ 9.03 (br s, 1H), 8.94 (s, 1H), 8.85 (s, 1H), 8.48 (d, *J* = 4.84 Hz, 1H), 8.34 9 (br, 1H), 8.08 (d, *J* = 7.96 Hz, 1H), 7.88 (d, *J* = 7.80 Hz, 2H), 7.49 (d, *J* = 7.64 Hz, 2H), 7.30 (d, *J* = 7.56 Hz, 2H), 7.25–7.23 (m, 3H), 7.18 (d, *J* = 4.72 Hz, 1H), 7.09–7.05 (m, 2H), 3.94 (s, 2H), 2.57 (m, 1H), 2.37 (s, 3H), 1.98–1.45 (m, 8H), 1.29–1.18 (m, 2H); HRMS: *m/z* 493.2718 [M + H]^+^.
  xiii)  **Compounds 1k-Boc and 1k.** Intermediate **5b** (46 mg, 0.14 mmol) reacted with **6g** (45 mg, 0.14 mmol) under Buchwald coupling conditions using Pd_2_(dba)_3_ (5 mg), XanthPhos (8 mg), and Cs_2_CO_3_ (66 mg) in 1,4-Dioxane (2 mL) overnight at 100°C to give **1k-Boc** (51 mg, 62%). MS of **1k-Boc**: *m/z* 592.33 [M + H]^+^.
Compound **1k-Boc** (40 mg, 0.068 mmol) was deprotected using 4 M HCl in dioxane/EtOAc (1:1, 2 mL) at RT. Solvents were removed under reduced pressure and worked up using CH_2_Cl_2_ and Aq. NaHCO_3_ solution to afford compound **1k** (30 mg, 89%) after purification. ^1^H NMR (600 MHz, CDCl_3_) of **1k**: δ 8.41 (d, *J* = 5.04 Hz, 1H), 8.31 (s, 1H), 8.04 (d, *J* = 7.96 Hz, 1H), 7.88 (d, *J* = 7.72 Hz, 3H), 7.78 (d, *J* = 7.64 Hz, 1H), 7.49–7.42 (m, 4H), 7.27–7.24 (m, 3H), 7.15 (d, *J* = 4.96 Hz, 1H), 7.03 (t, *J* = 7.24 Hz, 1H), 3.90 (s, 2H), 3.38 (m, 1H), 2.34 (s, 3H), 1–97–1.62 (m, 6H), 1.26–1.16 (m, 4H); HRMS: *m/z* 492.2772 [M + H]^+^.
xiv)  **Compound 1L.** Intermediate **5a** (100 mg, 0.29 mmol) underwent Buchwald coupling with **6h** (98 mg, 0.29 mmol) in the presence of Pd_2_(dba)_3_ (14 mg), XanthPhos (20 mg), and Cs_2_CO_3_ (142 mg) in 1,4-Dioxane (10 mL) as described above for IMTi-1. After the reaction mixture was stirred at 100°C overnight, usual work up and purification gave the title product **1L-Boc** (161 mg, 98%). ^1^HNMR (600 MHz, CDCl_3_) of **1L-Boc**: δ 9.04 (s, 1H), 8.95 (s, 1H), 8.88 (s, 1H), 8.50 (s, 2H), 8.10 (d, *J* = 6.0 Hz, 1H), 7.91 (d, *J* = 6.0 Hz, 2H), 7.37 (br t, J =, 2H), 7.30 (d, *J* = 6.0 Hz, 1H), 7.26 (d, *J* = 6.0 Hz, 1H), 7.20 (m, 2H), 4.18 (m, 1H), 2.82 (br, 2H), 2.40 (s, 3H), 2.08 (m, 1H), 1.82 (m, 1H), 1.69 (m, 2H), 1.61 (s, 3H), 1.49 (s, 6H), 1.48 (m, 2H); MS (ESI) *m/z* 564.28 [M]^+^.
Intermediate **1L-Boc** (90 mg, 0.16 mmol) in EtOAc (3 mL) was **Boc**-deprotected using 4 M HCl in Dioxane (2 mL) to give compound **1L** (70 mg, 94%) after usual work up using CH_2_Cl_2_ and NaHCO_3_ and filtration over a short bed of Silica gel. HRMS of **1L**: *m/z* 465.2401 [M + H]^+^.
xv)  **Compound 1m.** Intermediate **5b** (165 mg, 0.49 mmol) underwent Buchwald coupling with 6h (148 mg, 0.49 mmol) in the presence of Pd_2_(dba)_3_ (22 mg), XanthPhos (30 mg), and Cs_2_CO_3_ (235 mg) in 1,4-Dioxane (10 mL) as described above. After the reaction mixture was stirred at 100°C overnight, usual work up and purification gave the title product **1m-Boc** (265 mg, 87%). ^1^HNMR (600 MHz, CDCl_3_) of 1m-Boc: δ 8.50 (d, *J* = 6.0 Hz, 1H), 8.37 (s, 1H), 8.16 (d, *J* = 12.0 Hz, 1H), 7.93 (s, 1H), 7.89–7.86 (m, 3H), 7.545 (t, *J* = 6.0 Hz, 1H), 7.42 (d, *J* = 6.0 Hz, 1H), 7.32 (t, *J* = 12.0 Hz, 2H), 7.27 (d, *J* = 6.0 Hz, 1H), 7.22 (d, *J* = 6.0 Hz, 1H), 7.08 (t, *J* = 6.0 Hz, 1H), 6.96 (s, 1H), 4.18 (m, 1H), 2.81 (br, 2H), 2.40 (s, 3H), 2.08 (m, 1H), 1.81 (m, 1H), 1.68 (m, 2H), 1.59 (s, 3H), 1.51 (s, 6H), 1.48 (m, 2H); MS: *m/z* 564.29 [M + H]^+^.
Intermediate **1m-Boc** (95 mg, 0.17 mmol) was **Boc**-deprotected using 2 M HCl in Dioxane (2 mL) to give compound **1m** (75 mg, 95%) after usual work up using CH_2_Cl_2_ and NaHCO_3_ and filtration over a short bed of Silica gel. HRMS of **1m**: *m/z* 464.2459 [M + H]^+^.
xvi)  **Compound 1n.** NaCNBH_3_ (25 mg) and AcOH (50 µL) were added sequentially to a solution of amine **1L.HCl** (51 mg, 0.1 mmol) and paraformaldehyde (30 mg) in MeOH/2N Aq. KOH (10:1, 1.1 mL) at ice-water temperature and the reaction mixture was stirred at RT for another 8 h. Solvents were removed under reduced pressure, and the residue was suspended in CH_2_Cl_2_ and washed using water. Combined organic layers were dried using Na_2_SO_4_, filtered, and concentrated under reduced pressure. The residue was purified by Silica gel column to afford **1n** (40 mg, 83%). ^1^H NMR (600 MHz, CD_3_OD + CDCl_3_): δ 8.99 (br s, 1H), 8.94 (s, 1H), 8.45 (s, 1H), 8.02 (d, *J* = 6.0 Hz, 2H), 7.74 (d, *J* = 6.0 Hz, 1H), 7.49 (d, *J* = 6.0 Hz, 2H), 7.30–7.24 (m, 3H), 7.10 (t, *J* = 6.0 Hz, 1H), 3.49 (m, 2H), 3.10 (t, *J* = 12.0 Hz, 1H), 2.95 (t, *J* = 12.0 Hz, 1H), 2.87 (m, 1H), 2.81 (s, 3H), 2.34 (s, 3H), 2.10–1.64 (m, 4H); HRMS: *m/z* 479.2560 [M + H]^+^.
xvii)  **Compound 1o.** Reductive amination of amine **1m** (50 mg, 0.1 mmol) with paraformaldehyde (30 mg), NaCNBH_3_ (30 mg), and AcOH (50 µL) in MeOH/2N Aq. KOH (10:1, 1.1 mL) as described for **1n** afforded **1o** (32 mg, 67%). ^1^HNMR (600 MHz, CDCl_3_) of **1o**: δ 8.42 (d *J* = 6.0 Hz, 1H), 8.36 (s, 1H), 8.05 (d, *J* = 6.0 Hz, 1H), 7.87 (d, *J* = 6.0 Hz, 2H), 7.80 (d, *J* = 6.0 Hz, 1H), 7.47 (t, *J* = 6.0 Hz, 1H), 7.34 (d, *J* = 12.0 Hz, 2H), 7.265 (d, *J* = 6.0 Hz, 1H), 7.23 (d, *J* = 6.0 Hz, 1H), 7.16 (q, *J* = 6.0 Hz, 1H), 7.05 (t, *J* = 6.0 Hz, 1H), 3.29 (d, *J* = 12.0 Hz, 2H), 3.10 (t, *J* = 6.0 Hz, 1H), 2.66 (s, 3H), 2–63–2.54 (m, 2H), 2.34 (s, 3H), 2.03–1.92 (m, 3H), 1.65–1.61 (m, 1H); HRMS: *m/z* 478.2602 [M + H]^+^.
xviii) **Intermediate 5c.** Intermediate **4c** (156 mg, 0.5 mmol) underwent Buchwald coupling with 3-aminopyridine (50 mg) in the presence of Pd_2_(dba)_3_ (18 mg), XanthPhos (20 mg), and Cs_2_CO_3_ (250 mg) in 1,4-Dioxane (3 mL) by reacting the reaction mixture overnight at 100°C. Usual work up using CH2Cl2 and water and purification of the concentrated organic layers using Combi Flash afforded **5c**-**Boc** (160 mg, 86%). The latter product was treated with 4 M HCl in dioxane (2 mL) overnight, and solvents were removed to afford **5c** as HCl salt. MS of **5c**: *m/z* 264.12 [M + H]^+^.
xix)  **Compound 1p.** Prepared by amide formation between **5c.**HCl (30 mg, 0.1 mmol) and **6j** (20 mg, 0.1 mmol) using PyBOP (78 mg, 0.15 mmol) and DIPEA (60 µL) in DMF (250 µL) to afford **1p** (20 mg, 46%) after work up and purification by preparative TLC. ^1^H NMR (600 MHz, CDCl_3_): δ 8.49 (d, *J* = 6.0 Hz, 1H), 8.38 (s, 1H), 8.15 (d, *J* = 6.0 Hz, 1H), 7.99 (br s, 1H), 7.88–7.83 (m, 4H), 7.68 (d, *J* = 6.0 Hz, 1H), 7.53 (t, *J* = 6.0 Hz, 1H), 7.42–7.38 (m, 2H), 7.33 (d, *J* = 12.0 Hz, 1H), 7.26 (d, *J* = 6.0 Hz, 1H), 7.21 (d, *J* = 6.0 Hz, 1H), 7.08 (t, *J* = 6.0 Hz, 1H), 6.97 (s, 1H), 2.29 (q, *J* = 6.0 Hz, 2H), 2.61 (q, *J* = 6.0 Hz, 2H), 2.40 (s, 3H), 2.38 (s, 6H); HRMS: *m/z* 439.2256 [M + H]^+^. Purity (HPLC): >95%.
xx)  **Compound 1q** (Prepared by Suzuki reaction). To a degassed solution of intermediate **5a** (34 mg, 0.1 mmol) and boronic acid **7a** (35 mg, 0.15 mmol) in DMF (2 mL) and 2 M aq. K_2_CO_3_ solution (2 M, 0.2 mL) in a microwave vial was added Pd(PPh_3_)_4_ (11 mg) and the mixture was heated at 100°C for 30 min using microwave. The reaction mixture was diluted using water, extracted using EtOAc, and the combined organic layers concentrated under reduced pressure and the residue was purified by preparative TLC to afford **1q** (15 mg, 30%). ^1^H NMR (400 MHz, CDCl_3_): δ 9.18 (s, 1H), 8.92 (s, 1H), 8.56 (s, 1H), 8.51 (d, *J* = 5.04 Hz, 1H), 8.08 (d, *J* = 8.00 Hz, 1H), 7.63 (d, *J* = 7.88 Hz, 2H), 7.48 (d, *J* = 7.88 Hz, 2H), 7.25–7.21 (m, 4H), 7.09–7.05 (m, 1H), 3.49 (s, 2H), 2.37 (s, 3H), 2.32 (s, 6H); HRMS: *m/z* 396.2197 [M + H]^+^.
xxi)  **Compound 1r.** As described above for 1q, Suzuki reaction of **5a** (100 mg, 0.29 mmol) with **7b** (84 mg, 0.38 mmol) in DMF (2 mL) and aq. K_2_CO_3_ solution (2 M, 0.3 mL) in a microwave vial in the presence of Pd(PPh_3_)_4_ (20 mg) and heating the reaction mixture in microwave at 110°C for 30 min afforded **1r** (35 mg, 28%) after work up and purification using preparative TLC. ^1^H NMR (400 MHz, CDCl_3_) of **1r**: δ 9.14 (s, 1H), 8.92 (s, 1H), 8.52 (d, *J* = 4.52 Hz, 2H), 8.13 (d, *J* = 6.0 Hz, 1H), 7.60 (d, *J* = 8.56 Hz, 2H), 7.48 (d, *J* = 8.56 Hz, 1H), 7.25–7.21 (m, 2H), 7.10–6.96 (m, 3H), 3.34–3.23 (m, 8H), 1H), 2.81 (s, 3H), 2.34 (s, 3H), 2.10–1.64 (m, 4H); HRMS: *m/z* 437.2449 [M + H]^+^.

### 2.2 Synthesis of IMTi-2 and analogs 2a-b and IMTi-3

i)  **IMTi-2.** Buchwald coupling of intermediate **8** (54 mg, 0.24 mmol) with amine **9** (56 mg, 0.24 mmol) at 100°C overnight in the presence of Pd_2_(dba)_3_ (10 mg), XanthPhos (15 mg), and Cs_2_CO_3_ (140 mg) in 1,4-Dioxane (3 mL), as described above for IMTi-1, afforded intermediate **10** (41 mg, 42%). MS of **10**: *m/z* 409.16 [M]^+^.
Intermediate **10** (41 mg, 0.1 mmol) underwent reductive amination with 4-methylpiperazine (25 µL) using NaCNBH_3_ (65 mg) in dichloroethane (DCE) (3 mL) and AcOH (0.1 mL) as described for **1o** to give IMTi-2 (30 mg, 60%). ^1^H NMR (400 MHz, CDCl_3_+ CD_3_OD) of *IMTi*-2: δ 9.15 (s, 1H), 8.81 (s, 1H), 8.46 (d, *J* = 4.88 Hz, 1H), 8.35 (s, 1H), 8.27 (d, *J* = 6.88 Hz, 1H), 8.07 (d, *J* = 7.64 Hz, 2H), 7.85 (m, 1H), 7.57 (d, *J* = 8.0 Hz, 1H), 7.47 (d, *J* = 8.8 Hz, 1H), 7.45 (d, *J* = 8.0 Hz, 2H), 7.37 (m, 1H), 7.24 (d, *J* = 8.0 Hz, 1H), 7.15 (d, *J* = 4.96 Hz, 1H), 3.58 (s, 2H), 2.52 (br s, 8H), 2.33 (s, 6H); MS: *m/z* 494.2678 [M + H]^+^.
ii)  **Compounds 2a and 2b.** Aldehyde **8** (116 mg, 0.53 mmol) underwent reductive amination with neopentyl amine (130 µL) using NaCNBH_3_ (324 mg) in DCE (2 mL) and AcOH (0.1 mL) over 2 h. Reaction mixture was extracted using CH_2_Cl_2_, concentrated, and the residues taken in acetonitrile was stirred with Boc_2_O (300 mg) overnight to afford intermediate **11a** (165 mg, 80% in 2 steps). Subsequently, Amine **9** (27mg, 0.12 mmol) underwent Buchwald coupling with **11a** (46 mg, 0.12 mmol) in the presence of Pd_2_(dba)_3_ (4 mg), XanthPhos (7 mg), and Cs_2_CO_3_ (58 mg) in 1,4-Dioxane (3 mL) to afford **2a**-**Boc** (53 mg, 77%), and the latter product was deprotected using TFA in the presence of tri-isopropyl silane (TIPS) in CH_2_Cl_2_ giving **2a** (38 mg, 90%) after filtration using a short bed of silica gel column. MS of **2a**: *m/z* 481.27 [M + H]^+^.
Similarly, Aldehyde **8** (112 mg, 0.51 mmol) underwent reductive amination with cyclohexyl amine (100 µL) using NaCNBH_3_ (324 mg) in DCE (2 mL) and AcOH (0.1 mL) over 2 h, and the reaction mixture was extracted using CH_2_Cl_2_, concentrated, and the residues taken in acetonitrile was further reacted with Boc_2_O (300 mg) overnight to afford intermediate **11b** (175 mg, 85% in 2 steps). Next, amine **9** (24mg, 0.09 mmol) underwent Buchwald coupling with **11b** (36 mg, 0.09 mmol) in the presence of Pd_2_(dba)_3_ (4 mg), XanthPhos (6 mg), and Cs_2_CO_3_ (44 mg) in 1,4-Dioxane (2 mL) to afford **2b**-**Boc** (36 mg, 68%), and the latter product was deprotected using TFA in the presence of TIPS in CH_2_Cl_2_ giving **2b** (25 mg, 86%) after filtration using a short bed of silica gel column. MS of **2b**: *m/z* 493.27 [M + H]^+^.
iii)  **Intermediate 13.** To a solution of 3-aminopyridine (1 equiv.) and DIEA (3 equiv.) in dry THF (5 mL/mmol) was added 4-chloromethylbenzoyl chloride (1.2 equiv.) at room temperature and the resulting mixture was stirred for 16 h and evaporated under reduced pressure. The residues were worked up using CH_2_Cl_2_ and Aq. NaHCO_3_ and purified to afford intermediate **12.** MS of **12**: *m/z* 247.06/249.06 [M + H]^+^.
A solution of intermediate **12** (1 equiv.), N-Boc-piperidine (1 equiv.), and DIEA (3 equiv.) in dry THF (5 mL/mmol) was heated at 90°C for 16 h. Reaction mixture was worked-up using water and CH_2_Cl_2_, and the combined organic layers concentrated under reduced pressure and chromatographed over Silica gel using CH_2_Cl_2_-MeOH-aq. NH_3_ to afford **13**-**Boc**. The latter underwent Boc deprotection using methanolic HCl to afford **13**. MS: *m/z* 297.17 [M + H]^+^.
iv)  **Intermediate 14.** 2-Aminopyrimidine (95 mg, 1.0 mmol) underwent Buchwald coupling with 3-bromo-4-methylbenzaldehyde (199 mg, 1.0 mmol) in the presence of Pd_2_(dba)_3_ (45 mg), XanthPhos (60 mg), and Cs_2_CO_3_ (489 mg) in 1,4-Dioxane (10 mL) overnight at 100°C to give intermediate **14** (206 mg, 96%). MS of **14**: *m/z* 214.09 [M + H]^+^.
v)  **IMTi-3.** A solution of **13** (40 mg, 0.13 mmol) and **14** (28 mg, 0.13 mmol) in dichloroethane (5 mL) and AcOH (0.2 mL) was added Na(OAc)_3_BH (100 mg, 0.47 mmol) in portions at 0°C. After the reaction mixture was stirred overnight at room temperature, usual work up using methylene chloride and Aq. NaHCO_3_ solution and purification over Silica gel gave IMTi-3 (34 mg, 50%). ^1^H NMR (400 MHz, CDCl_3_+ CD_3_OD) of IMTi-3: δ 8.68 (s, 1H), 8.37 (s, 1H, and d, *J* = 4.6 Hz, 2H), 8.30 (s, 2H), 7.83 (d, *J* = 7.13 Hz, 2H), 7.82 (s, 1H), 7.43 (d, *J* = 7.88 Hz, 2H), 7.31 (dd, *J* = 7.96, 4.6 Hz, 1H), 7.17 (d, *J* = 7.64 Hz, 1H), 7.01 (d, *J* = 7.48 Hz, 1H), 6.89 (s, 1H), 6.69 (t, *J* = 4.72 Hz, 1H), 3.57 (s, 4H), 3.53 (s, 4H), 2.29 (s, 3H); HRMS: *m/z* 494.2672 [M + H]^+^.

### 2.3 Screening and evaluation of IMTi’s and analogs

N2a695 cells were used to screen all new compounds and in the follow-up studies with compounds found active in the preliminary screen. In a typical experiment, 6-well tissue culture plates (Corning) were seeded at 4.0 × 10^5^–4.5 × 10^5^ N2a695 cells/mL, 2 mL/well for overnight incubation. When cells were >95% confluent, media were exchanged with fresh media containing 10 µM solutions of compounds or DMSO carrier alone and cells were incubated at 37°C in 5% CO_2_ for 5 h. Culture media were collected and soluble Aβ concentrations in the media were determined by ELISA or MSD plates for human Aβ peptides as per manufacturer instructions. Signals for Aβ were measured using Perkin Elmer Envision and SQ120 MSD ELISA reader. Follow-up studies with N2a695 cells were performed similarly.

### 2.4 Effects of IMT and IMTi’s on APP metabolism

N2a695 cells were treated with compounds for 5 h as described above, and media were aspirated out (or collected for determination of Aβ levels). Cells were scraped in cold Dulbecco’s PBS buffer (1 mL) containing mini EDTA-free protease inhibitor (Roche) and centrifuged for 1 min at 13,000 rpm at 4°C to form a cell pellet. The buffer was aspirated, and the cell pellets were lysed in 3% SDS plus protease inhibitor cocktail by sonication on ice for two rounds of 20 s on a low setting. Protein concentrations were measured using the Pierce BCA Protein Assay (Thermo Fisher) kit in accordance with the manufacturer’s instructions.

To perform WBs, N2a695 cell lysates from **1a** and analogs-treated samples were run on a 10%–20% or a 16.5% Tris-Tricine gel (Criterion) and electro transferred to PVDF membranes (EMD Millipore) overnight at 30 V. PVDF membranes were incubated in PBS containing 0.25% glutaraldehyde (Sigma) for 30 min after electro transference, blocked for 30 min in milk PBST, incubated with primary antibody RU369 for 1 h at room temperature followed by washing and incubation with an HRP-linked secondary antibody and detected with enhanced chemiluminescence ECL reagents. WB images were analyzed using ImageJ to quantify the prominent bands.

To determine effects of compounds on BACE1 vs. GS inhibition, we used N2a cells transiently transfected with full length APP (APP-FL) or with APP99 (APP-βCTF) as described previously (Netzer et al., 2017; Sun et al., 2019). After 48 h, media were removed and fresh media containing compound **1a** and analogs were added. Following 5 h of incubation, cell supernatants were collected, and analyzed using MSD-ELISA for Aβ and sAPPα and western blot for sAPPβ.

### 2.5 *In vivo* brain permeability and retention of IMT analogs

All procedures involving animals were approved by The Rockefeller University Institutional Animal Care and Use Committee and were in accordance with the National Institutes of Health guidelines. Mesylate salts of the isomeric IMT analogs (1 or 3 mg/mL in water, 125 μL, 50 mg/kg) were administered intraperitoneally (i.p.) or through oral gavage to 8 weeks old C57BL/6J WT mice (Sun et al., 2019). Mice were euthanized 4 h post drug administration and brain hemispheres and plasma were harvested and collected in pre-weighted tubes and snap-frozen in liquid nitrogen. To measure brain and plasma concentrations of the specific compounds, mouse brain tissue was homogenized and extracted using Ethanol, and plasma samples were extracted using Acetonitrile. Concentration of the drug and metabolites in brain and in plasma was determined by LC-MS/MS analysis.

### 2.6 Drug extraction from brain

After tubes were weighed to calculate brain weight and thawed to room temperature, 1 mL of EtOH (200 Proof) was added to the microcentrifuge tubes containing the harvested right brain hemispheres. 10 μL of 1 µM internal standard (ABG190, a synthetic analog of 1a) was added to each tube and samples were sonicated to homogeneity (∼2 min). Tubes were shaken at 40 min at room temperature (1K RPM) and centrifuged for 8 min at 13K RPM. The supernatant (0.9 mL) was transferred to a new collection tube and 0.5 mL EtOH was added to the pellet for a second round of extraction as described above. 600 μL of the supernatant was combined with the first collection before samples were submitted for LCMS-MS analysis (Sun et al., 2019).

### 2.7 Drug extraction from blood

300 μL of acetonitrile was added to collected blood samples. 10 μL of 1 µM internal standard (ABG190) was added to each tube and samples were sonicated to homogeneity (∼2 min). Tubes were contributed at 13 K RPM for 9 min 300 μL of the supernatant was collected and combined with 500 µL of 5 mM ammonium formate before samples were submitted for LCMS-MS analysis (Sun et al., 2019).

### 2.8 *In vitro* kinase activity assay

The assay was performed by Luceome Biotechnologies, LLC. Typically, 10 mM stock solutions of the compounds were diluted in DMSO to a concentration of 250 μM. Prior to initiating the assay, all test compounds were evaluated for false positive against split-luciferase (Jester et al., 2010). For kinase assays, each Cfluc-Kinase was translated along with Fos-Nfluc using a cell-free system (rabbit reticulocyte lysate) at 30°C for 90 min 24 μL aliquot of this lysate containing either 1 μL of DMSO (for no-inhibitor control) or compound solution in DMSO (10 μM final concentration) was incubated for 30 min at room temperature followed by 1 h in presence of a kinase specific probe. 80 μL of luciferin assay reagent was added to each solution and luminescence was immediately measured on a luminometer. The percent Inhibition was calculated using the following equation: % Inhibition = (ALUcontrol–ALUsamplex 100)/ALUcontrol.

## 3 Results

### 3.1 Chemistry

**SCHEME 1:**
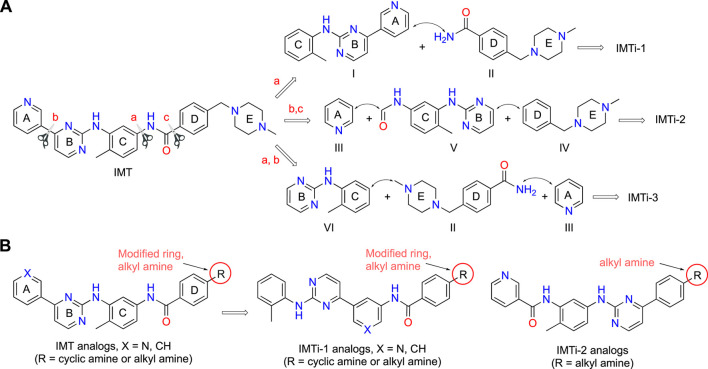
Design of IMTi-1 – 3 and analogs. **(A)** Shown are hypothetical fragmentation of IMT involving (a) C-N bond or (b, c) C-C bond cleavage giving fragments **I**-**VI**, and re-assembly of these fragments to afford IMTi-1 – 3. Note: fragment **II** is common for both IMTi-1 and 3, and **III** for IMTi-2 and 3. Key: Scissor sign, site of C-C or C-N bond cleavage for fragmentation; double arrow, C-C or C-N bond connection for re-assembly of the molecules. **(B)** General structure of IMT analogs described previously (Sun et al., 2019) and of IMTi-1’s and 2’s designed here.

IMT isomers, IMTi-1 – 3, possess all five rings and the chemical functions that broadly match the parent compound (Figure 1). We designed these isomers by making one or two hypothetical fragmentations across C-N and C-C bonds and re-joining the resulting fragments through other ring(s) and keeping the functionalities similar to IMT, as outlined in Scheme 1A. Here, cleavage sites shown by “scissor” are evident in IMT at ‘a-c’ and the double arrows shown connect **I** and **II**; **III**, **V** and **IV**; and **VI**, **II** and **III** to give IMTi-1, IMTi-2, and IMTi-3, respectively. Similarly, we designed IMTi-1 and 2 analogs based on the previously described IMT analogs, in that ‘E’ ring has been modified with R = various alkyl and cycloalkyl amines and ‘A’ ring with phenyl and substituted phenyls besides pyridine (Scheme 1B) (Sun et al., 2019).

**SCHEME 2:**
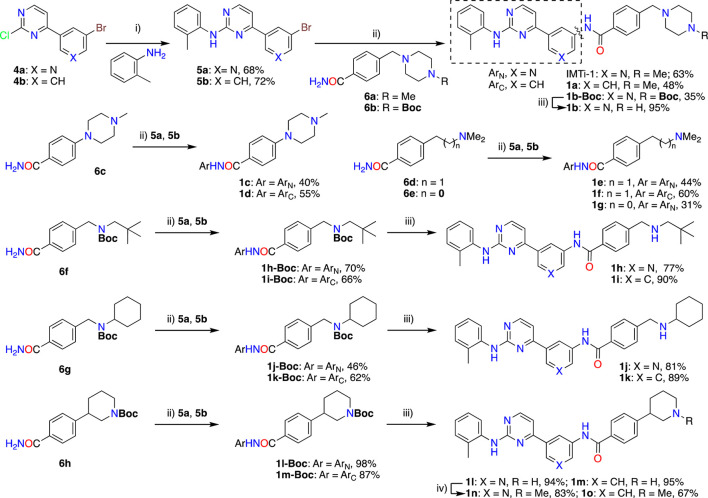
Synthesis of *IMTi*-1 and analogs 1a-o Key: i) 3N HCl, Dioxane. microwave, 100°C, 2 h ii) Pd_2_(dba)_3_, XanthPhos, Cs_2_CO_3_, 1,4-Dioxane, microwave, 100°C. iii) 4M HCl in dioxane, EtOAc, RT, 2 h iv) CH_2_O, NaCNBH_3_, DCE, 0°C - RT, 16 h.

**SCHEME 3:**
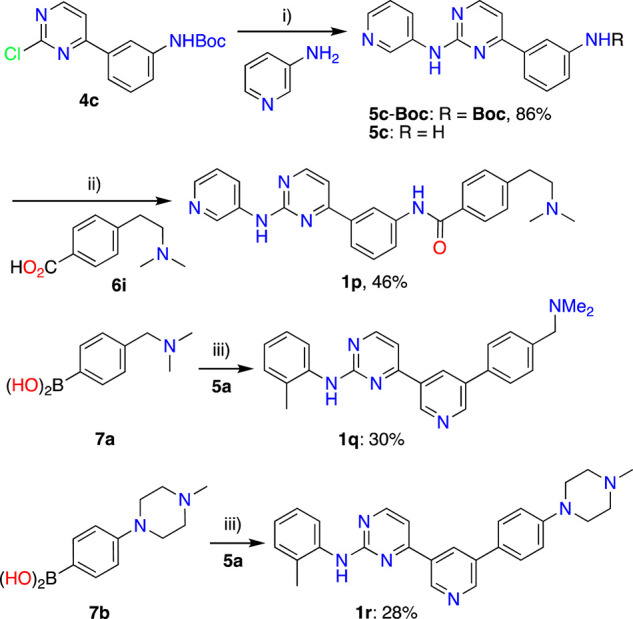
Synthesis of *IMTi*-1 analogs 1p-r. Key: i) Pd_2_(dba)_3_, XanthPhos, Cs_2_CO_3_, 1,4-Dioxane, microwave, 100°C; 4M HCl in dioxane, EtOAc, RT, 2 h ii) PyBOP, DIPEA, DMF, RT, 16 h iii) Pd(PPh_3_)_4_, aq. K_2_CO_3_, 1,4-Dioxane, microwave, 100°C, 2 h.

**Synthesis of IMTi-1 – 3 and analogs.** We prepared IMTi-1 and its analogs **1a**-**1r** using the readily available intermediates, as outlined in Schemes 2, 3. First, to prepare IMTi-1, intermediate **4a** was reacted with o-toluidine and the resulting product **5a** underwent Buchwald coupling (Ruiz-Castillo and Buchwald, 2016) with amide **6a** (Scheme 2). Similarly, intermediate **4b** reacted with o-toluidine to give **5b**, and both **5a** and **5b** underwent Buchwald coupling (Ruiz-Castillo and Buchwald, 2016) with various amides **6a**-**h** giving products **1a**-**m**, several after Boc deprotection as needed. Analogs **1n** and **1o** were prepared by reaction of **1l** and **1m** with formaldehyde under the reductive amination conditions using NaCNBH_3_. The analog **1p** was obtained by reacting **4c** with 3-aminopyridine, followed by Boc-deprotection giving amine **5c** and reacting the latter with acid **6i** (Scheme 3). Finally, Analog **1q** and **1r** were prepared by Suzuki coupling (Miyaura and Suzuki, 1995) of **5a** with boronic acids, **7a** and **7b** (Scheme 3).

**SCHEME 4:**
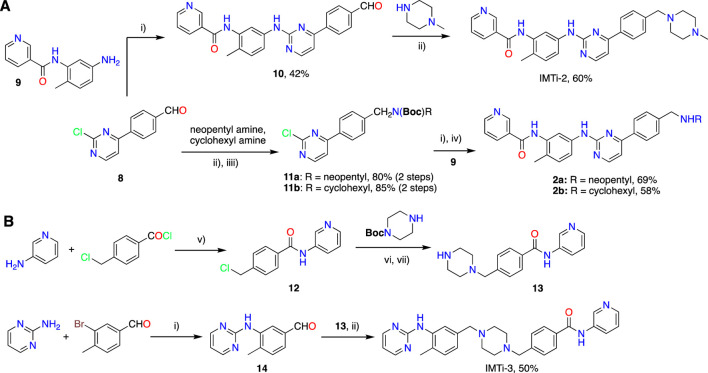
Synthesis of **(A)** IMTi-2 analogs, and **(B)** IMTi-3. Key: i) Pd(dba)_3_, XanthPhos, Cs_2_CO_3_, 1,4-Dioxane, microwave, 100°C, 2 h ii) Na(OAc)_3_BH, DCE, AcOH. iii) Boc_2_O, ACN. iv) TFA, TIPS, CH_2_Cl_2_, 0°C-RT, 2 h v) DIEA, THF, RT, 3 h vi) DIEA, THF, 90°C, 2 h vii) 4M HCl in dioxane, EtOAc, RT, 2 h.

Next, we prepared IMTi-2 and its analogs **2a**-**b** using intermediates **8** and **9**, as described in Scheme 4A. Intermediates **8** and **9** reacted together under the Buchwald coupling conditions affording **10**, which underwent reductive amination with N-methylpiperazine to give IMTi-2. Alternatively, intermediate **8** underwent reductive amination with cyclohexyl amine and neopentyl amine and Boc-protection of the resulting amines to give intermediates **11a** and **11b**, which reacted with intermediate **9** under the Buchwald coupling conditions, followed by Boc-deprotection to give analogs **2a**-**b**. Finally, to prepare IMTi-3, we prepared intermediate **13** by reacting 4-chloro-mthylbenzoyl chloride with 3-aminopyridine 3-amino-pyridine and the resulting product **12** with N-Boc-piperazine followed by N-deprotection, and intermediate **14** by reacting 3-bromo-4-methylbenzaldehyde with 2-amino-pyrimidine under Buchwald conditions. Subsequently, we coupled intermediates **13** and **14** together under the reductive amination conditions using NaCNBH_4_ to give the title IMTi-3 (Scheme 4B) (Afanasyev et al., 2019).

### 3.2 Structural diversity

As described above, many IMT analogs, in that either A ring changed to substituted benzene ring or E ring to cycloalkyl amines or alkyl amines, were prepared previously and evaluated (Sun et al., 2019). The majority of IMTi-1 analogs, including **1a**-**1p** and both analogs of IMTi-2, i.e., **2a** and **2b**, differ from one-another in ring ‘A’ and/or in ‘E’ and possess fragments containing ‘D’ and ‘E’ rings previously prepared in IMT analogs (Sun et al., 2019). New IMTi-1 analogs contain piperazine ring, a cyclic amine or piperidine ring connected through C-C or C-N bond to ring D, while all other IMTi-1 and both IMTi-2 analogs possess a substituted alkylamine instead of the ring E. These modifications improved APP processing activity in IMT analogs (Sun et al., 2019). There was no additional difference between two analogs, **2a** and **2b**, of IMTi-2. Thus, all 18 analogs of IMTi-1 possess rings ‘A-D’ and their arrangement is similar with three exceptions. 1) Seven compounds possess 1,3-substituted benzene and the remaining 11 analogs contain 3,5-substituted pyridine (Py) as the middle ring ‘A’, 2) The first ring from the left (ring ‘C’) in 1 analog, **1p**, is 3-aminopyridine instead of o-toluidine in all remaining 17 compounds. 3) Analogs **1q** and **1r** do not possess the ‘amide group’ that connects the middle ring ‘A’ to the 4th ring ‘D’.

### 3.3 Evaluation

**FIGURE 2:**
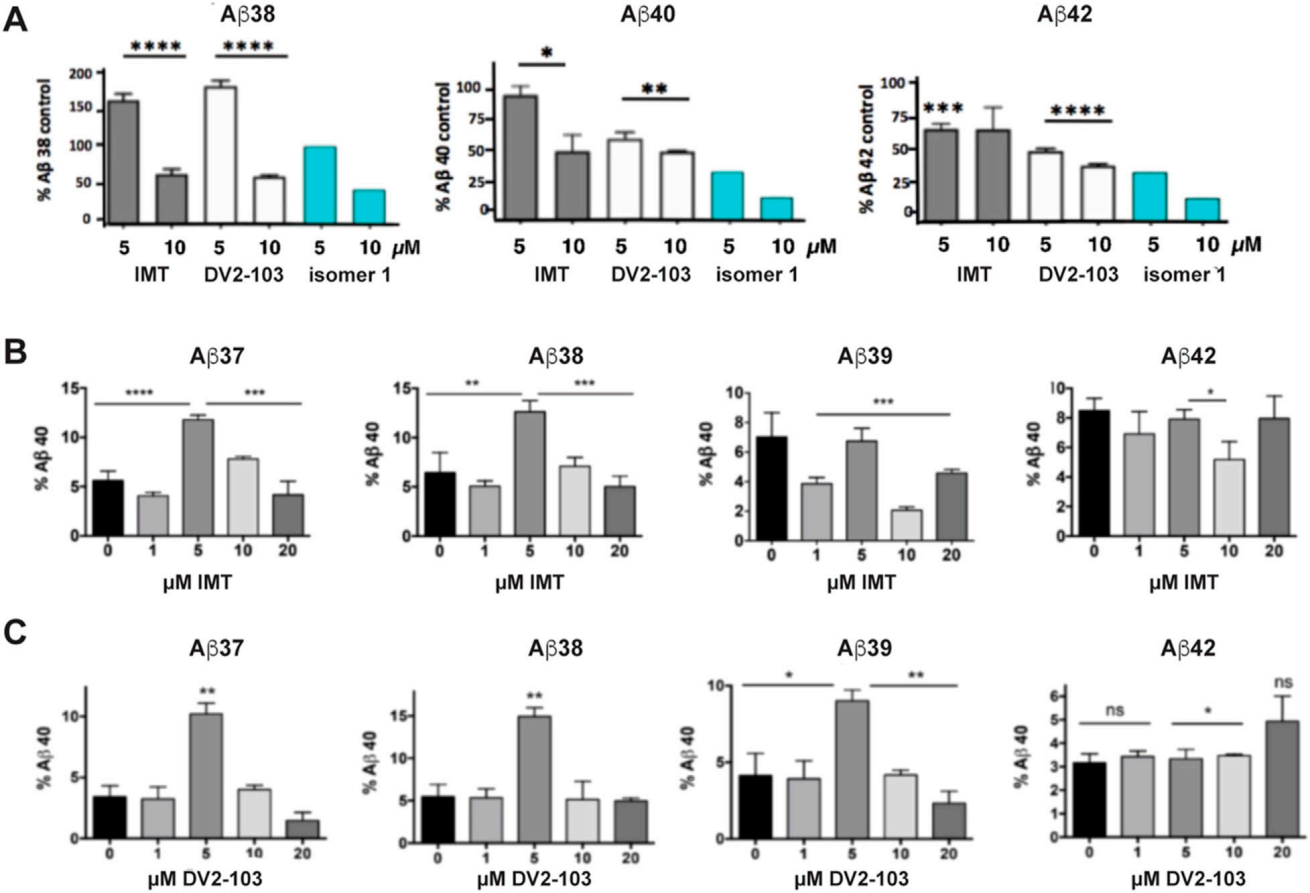
IMT, DV2-103, and IMT isomer **1** are γ-secretase modulators. **(A)** N2a695 cells incubated with IMT, DV2-103 and IMT isomer 1 lower levels of Aβ40 and 42 more than Aβ38, especially at a drug concentration of 5 μM, as measured by ELISA. Means differ significantly for IMT and DV2-103 compared to DMSO controls, N = 3 × 3. Data for Isomer 1 are from a representative sample. Differences between means for **(A)** are analyzed by One-way Anova for IMT and DV2-103 treated cells. Differences among means comparing 5 μM IMT and DMSO controls **(B, C)** are analyzed by Student’s T test (S.E.M.).

Previously, we showed that two chemically distinct compounds, IMT and DV2-103 lower Aβ production primarily by reducing BACE processing of APP (Netzer et al., 2017). Similarly, numerous analogs of IMT also lowered Aβ production by reducing BACE processing of APP (Sun et al., 2019). In the present study, before evaluating new analogs, we further examined the effects of these compounds on γ-secretase catalyzed Aβ formation and compared these to the more radically isomeric analogs of IMT. Our results show that IMT, DV2-103, and *IMTi*-1 are γ-secretase modulators; i.e., these compounds favor production or inhibition of different lengths of Aβ peptides (differing in their C-termini). Specifically, we exposed N2a695 cells to increasing concentrations of each compound and measured the production of Aβ38, 40, and 42. IMT, DV2-103 and IMTi-**1** (Figure 2A) inhibit the formation of Aβ38 least, compared to Aβ40 and 42, and even boost levels of Aβ38 above controls at a drug concentration of 5 μM. Remarkably, this occurs for all Aβ peptides tested shorter than 40 amino acids (Figure 2C). Moreover, for some compounds that modulate γ-secretase activity most conspicuously at 5 μM*,* this effect vanishes at 10 μM, relative to controls (Figure 2B, C).

**FIGURE 3:**
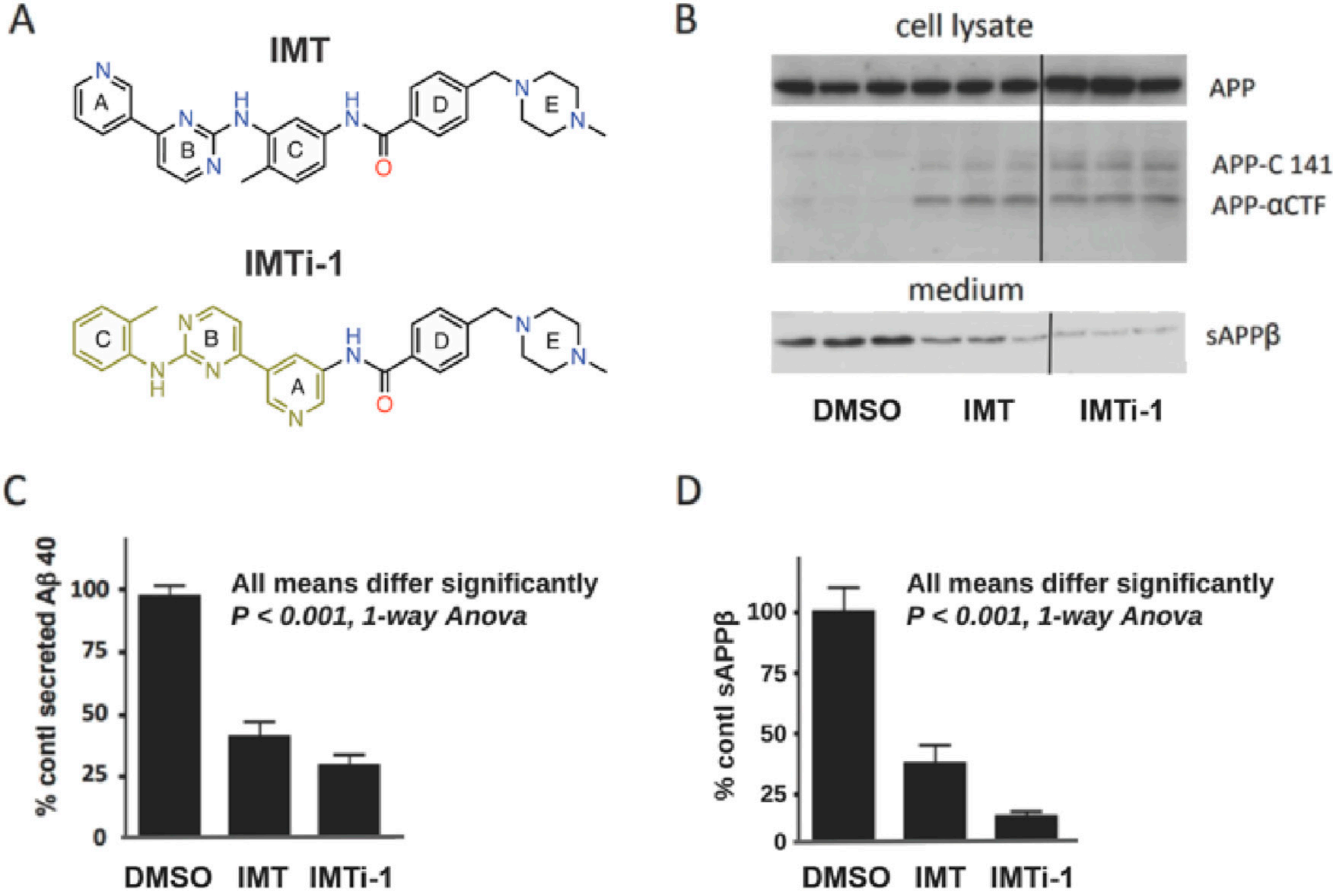
A Major structural change in IMT results in IMT isomer 1 (IMTi-1), which retains IMT’s Aβ-reducing effect and its reduction of BACE processing of APP in N2a 695 cells. **(A)** Structures of IMT and isomer-1, IMTi-1. **(B)** Western blots of N2a cell lysates (upper) and cell media (lower) from experiments using IMT and IMTi-1 probed with antibody RU369 (anti-C terminal APP) and RU anti-C-terminal sAPPβ (bottom), respectively. Each western blot panel shows lanes from a single gel. However, the three lanes at the right of each, which refer to incubation with IMTi-1, are from a different part of the same gel. **(C)** Quantification of secreted Aβ40 in N2a cells incubated with IMT or IMTi-1, One-way Anova, *p* < 0.001. **(D)** Quantification of sAPPβ levels. One-way Anova, *p* < 0.001.

With new IMTi’s in hand, we first evaluated and compared the effects of IMT and IMTi-1 (Figure 3A) on APP processing in N2a695 cells using the methods described above and in our prior reports (Netzer et al., 2017; Sun et al., 2019). We used both compounds at 10 µM and analyzed cell supernatants using anti- Aβ40 ELISA to show that IMTi-1 reduced Aβ40 levels more strongly than IMT (Figure 3C). We further determine the sAPPβ levels in cell supernatants by performing western blotting (WB) experiments and probing the WB membranes using antibody RU anti-C-terminal sAPPβ (Figure 3B, bottom, and Figure 3D), and APP metabolites in cell lysates using antibody RU369 (anti-C terminal APP) (Figure 3B, upper). Similarly, we tested the effects of IMTi-2 and 3 on Aβ production in N2a695 cells to find that both IMTi-2 and IMTi-3 inhibited Aβ40 production weakly (Aβ40 levels: 68% for IMTi-2 and 93% for IMTi-3 at 10 µM concentration) compared to both IMT and IMTi-1.

Similarly, we evaluated all IMTi-1 and IMTi-2 analogs, including the **Boc**-protected compounds, using N2a695 cells. We found most **Boc**-protected compounds showed little or no inhibition of Aβ production (Supplementary Figure S1) under the above conditions. In fact, two **Boc** compounds, **1i**-Boc and **1k**-Boc, showed an increase in Aβ production, whereas several IMTi-1 analogs showed superior inhibitory effects compared to IMT on Aβ production (Supplementary Figure S1). To further examine the activities of the active analogs and whether IMTi’s has any bias on amyloidogenic vs. nonamyloidogenic cleavage of APP, we retested dozens of IMTi-1 and 2 analogs, including several found active in the screening assay and some tested for the first time using N2a695 cells as above, and performed MSD ELISA of the conditioned media to measure Aβ40, Aβ38, and Aβ42 peptides simultaneously. We found that most IMTi-1 analogs favored nonamyloidogenic cleavage of APP at both 10 and 5 µM concentrations and reduced production of Aβ40 and Aβ42 greater than Aβ38 peptide (Table 1). This indicates that IMTi-1 and analogs modulate γ-secretase cleavage of C-terminal APP since the differences in lengths of these peptides is determined by γ-secretase according to differences in utilization of the APP γ-secretase cleavage sites (Hur, 2022).

**TABLE 1:** Isomeric IMT analogs are γ-secretase modulators^a^.

Comp. ID	% Aβ38, 40, 42 of DMSO ctrl at 10 (5 µM) conc	Comp. ID	% Aβ38, 40, 42 of DMSO ctrl at 10 (5 µM) conc
**1a**	86, 40, 35 (105, 57, 49)	**1b**	61, 34, 33 (95, 56, 53)
**1d**	22, 14, 11 (60, 34, 28)	**1e**	91, 46, 44 (99, 57, 54)
**1f**	80, 29, 26 (113, 52, 45)	**1h**	80, 43, 43 (106, 64, 58)
**1i**	85, 41, 46 (106, 56, 58)	**1j**	123, 73, 65 (112, 83, 75)
**1k**	92, 38, 38 (120, 60, 56)	**1L**	52, 21, 17 (107, 48, 43)
**1n**	95, 42, 38 (112, 63, 56)	**1p**	24, 18, 15 (58, 41, 38)
**2b**	74, 71, 74 (93, 90, 88)		

^a^ The screening experiment was performed for Aβ40, 38 and 42 in cell supernatants of N2a695 cells using MSD ELISA., Drug concentrations are 10 μM or 5 μM. Aβ values are expressed as percentages of control Aβ38, 40, and 42, respectively. Results for the IMTi-1, derivatives shown here were obtained from a single experiment.

**FIGURE 4:**
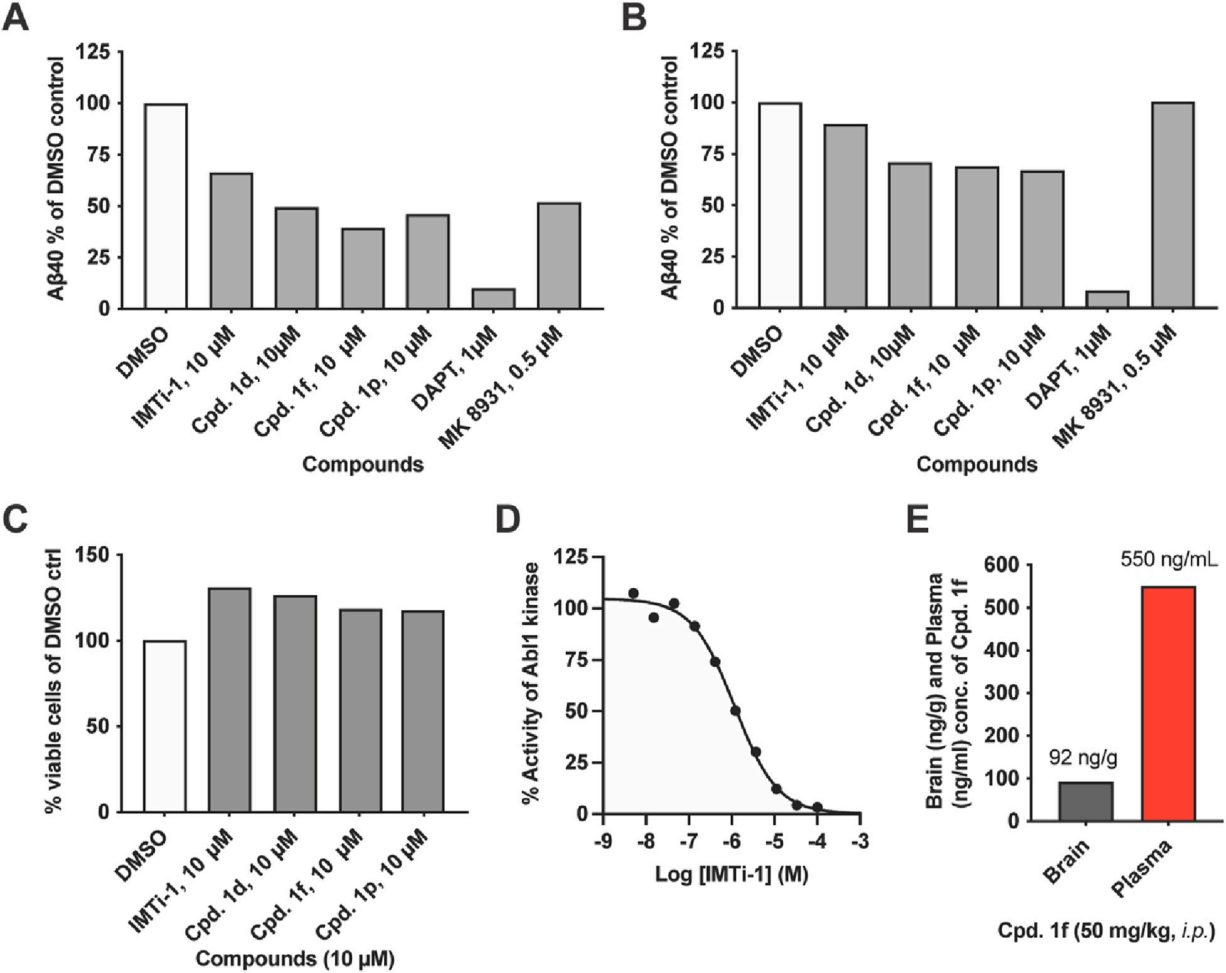
IMT isomer **1** (IMTi-1) and its analogs **1d**, **1f** and **1p** lower BACE and γ-secretase cleavage of APP and lower levels of Aβ in N2a cells transiently transfected with APP 695 (left graph) or APP C99 (right graph): **(A)** full length APP695 (APP-FL) or **(B)** APP C99 (β-CTF). **(C)** Percentage of viable wild-type (WT) N2a cells upon treatment with IMT isomers **1**, cpd. **1d**, cpd. **1f** and cpd**. 1p** compared to DMSO control under the same conditions used to test Aβ production. **(D)** Effects of IMTi-1 on Abl1 kinase *in vitro*. **(E)** Brain and plasma concentrations of IMTi-1 and cpd. **1f** in 2 months old WT mice 4 h after i. p. injection of 50 mg/kg of each drug. Data for A-C are from representative samples.

We focused on *IMTi*-1 and its analogs **1d**, **1f** and **1p**. All these analogs showed superior activities among all. To test whether these compounds lower Aβ levels by affecting the BACE and/or γ-secretase cleavages, we transfected wild-type (wt) cells with APP-FL and APP βCTF (C99), respectively, and incubated the cells with *IMTi*-1 (isomer) and analogs **1d**, **1f** and **1p**. We used BACE inhibitor, MK8931, and γ-secretase inhibitor, DAPT, as controls and performed the experiments and processed the results as described previously (Sun et al., 2019). The results shown in Figures 4A, B revealed that all 4 compounds reduced β- and γ-cleavages of APP similarly to IMT. There were reductions in Aβ production in both cases, but more so in cells transfected with full-length APP indicating that these compounds, like IMT (Netzer et al., 2017), reduce both BACE and γ-secretase cleavages of APP but that attenuation of BACE processing accounted for the greater part of Aβ reduction (Figures 4A, B). None of these compounds showed any toxicity to N2a695 cells at 10 µM concentration (Figure 4C) under the experimental conditions used for the Aβ assay.

IMT inhibits Abl1 kinase with low nanomolar affinity (Buchdunger et al., 1996). Earlier, we prepared and evaluated numerous IMT analogs to find that many of these analogs reduced Aβ levels in cells similarly to IMT, while inhibiting Abl kinase less potently, compared to IMT. In other words, there is not a good correlation between the Abl kinase inhibitory activity vs. the Aβ lowering effects in cells contacted with the IMT analogs. We have evaluated IMTi-1 to find that it inhibits Abl kinase less potently (IC_50_: 1.172 µM) (Figure 4D) than IMT IC_50_: 0.038 µM) (Buchdunger et al., 1996), while it reduced Aβ levels more potently than IMT (Figure 3C). This result further reinforces our prior observation that there is no or little connection between the Aβ-lowering activity of IMT and its inhibition of Abl1 kinase (Netzer et al., 2003). Finally, we tested the brain permeability of compound **1f** by administering it to 2 months old mice. Plasma and brain tissue were collected 4 h post drug administration, and LC-MS/MS analysis of the acetonitrile and ethanol extracts was used to measure drug concentration. Compound **1f** possesses similarity to IMT isomer-**1a** and is isomeric to an ABG-179 (Sun et al., 2019) analog that possessed a benzene instead of the pyridine (A) ring (see: Figure 1 for the ring numbering). Earlier, we have shown that ABG-179 possesses superior brain exposure compared to IMT and reduced both Aβ40 and 42 levels significantly in AD mice when delivered acutely for 5 days (Sun et al., 2019). Now, we have found that isomer **1f** also possesses high brain exposure (Figure 4E) and that is comparable to ABG-179 based on the results of our prior studies (Sun et al., 2019).

## 4 Discussion

Based on the number and variety of chemically distinct compounds that produce the same biochemical effects on APP metabolism (Netzer et al., 2017) and that all active compounds are active at low micromolar concentration, we postulated that IMT, DV2-103 and their analogs are likely to produce their effects on APP metabolism by virtue of their physical rather than stereological properties. For example, physical properties would include acting as a weak base that would cause these molecules to be lysosomotropic. We came to this conclusion by showing that the effects of IMT and DV2-103 on APP metabolism are dependent on acidified lysosomes (Netzer et al., 2017), and with the knowledge that IMT is strongly lysosomotropic we hypothesized that IMT, DV2-103 and their active derivatives might bind to a polyspecific receptor where binding is less dependent on structural and electrostatic complementarity. However, our subsequent studies with IMT, IMTi-1 – 3 and the analogs of IMTi-1 provide a more complex picture.

In our current study, we designed IMT isomers, IMTi-1 – 3, each possessing a distinctly unique pharmacophore, yet maintaining IMT’s physical property as a weak base (Supplementary Table S1), determined through quantitative structure property relationship (QSPR) analysis (Li et al., 2018). The weakly basic property of IMT is necessary for its sequestration in lysosomes through ion trapping (Burger et al., 2015). In other words, IMT is lysosomotropic, as are IMTi-1 –3 (Netzer et al., 2017). However, IMTi-3 was found inactive in Aβ production assays, while IMT and IMTi-1 and 2 were active.

Interestingly, we found that IMT, IMTi-1, and DV2-103 show concentration dependent modulation of γ-secretase as tested in Aβ production assay in N2a695 cells (Figure 2). Moreover, by evaluating novel IMTi-1 and 2 analogs, it became evident that a subset of these analogs recapitulated IMT’s APP phenotype. Additionally, we showed that IMT, DV2-103, and the IMTi-1 isomers tested in this study are modulators of γ-secretase by virtue of the observation that their Aβ-lowering potency differentially affects Aβ peptide lengths depending on drug concentration. Remarkably, Aβ1-42 production is lowered at 5 μM drug concentrations, while Aβ1-38 production is inhibited least and, in some cases, raised. This is important because heightened production of Aβ38 has been considered benign, and more recently therapeutic (Cullen et al., 2022), while lowered production of Aβ42 is considered therapeutic; in either case, a decrease in Aβ peptide aggregation may occur.

IMTi-1 inhibited Abl kinase activity with over a 100-fold reduction in potency compared to previously published reports of IMT (Buchdunger et al., 1996). Although we had compared the relative effects of γ-secretase and BACE modulation of Aβ generation in cells, we could not rule out that the lowering of Aβ and sAPPβ was not a result of IMT’s effect of stimulating autophagy (Drullion et al., 2012), since autophagy was previously shown to accelerate lysosomal degradation of APP-βCTF and Aβ(Tian et al., 2011). Further examination of the structures and activities of IMTi-1 and analogs compared to IMT and similar analogs (depicted by ‘R’ in Scheme 1B) revealed that both classes of active compounds possessed similar ‘R’ groups. Yet, unlike IMT and its analogs, all active IMT-1 analogs behaved like g-secretase modulators, thereby providing a new pharmacophore for development of anti-Aβ therapy.

## 5 Conclusion

In summary, we suggest that the effect of IMT and related drugs on APP metabolism occurs through a mechanism that is, to a great extent, determined by physicochemical and structural properties of the drug molecules and is less dependent on similarities in stereochemical structure. Future studies may wish to focus on trafficking of full-length APP to determine whether these drugs affect APP trafficking by translocation of APP to lysosomes and away from amyloidogenic processing by BACE and γ-secretase. The fact that many of these compounds (structurally related or not) are γ-secretase modulators may also be consistent with a mechanism involving altered trafficking of APP that could affect the specificity of γ-secretase cleavage sites on APP during the formation of Aβ peptides.

## Data Availability

The original contributions presented in the study are included in the article/Supplementary Material; further inquiries can be directed to the corresponding authors.
