# Supplementary material for: Stretching the structural envelope of imatinib to reduce β-amyloid production by modulating both β- and γ-secretase cleavages of APP
Source: Front Chem. 2024 Oct 8;12:1381205. doi: 10.3389/fchem.2024.1381205 (PMC11493595; doi:10.3389/fchem.2024.1381205)
Supplement: Supplementary file 1 [file DataSheet1.PDF]

Stretching the structural envelope of imatinib to reduce β-amyloid production by modulating both β- and γ-secretase cleavages of APP

William J. Netzer[1,*], Anjana Sinha[1,*], Mondana Ghias[1], Emily Chang[1], Katherina Gindinova[1], Emily Mui[1], Ji-Seon Seo[1], Subhash C. Sinha[1,2,*]

[1]Laboratory of Molecular and Cellular Neuroscience, The Rockefeller University, New York, NY 10065. [2]Appel Alzheimer's Disease Research Institute, Feil Family Brain and Mind Research Institute, Weill Cornell Medicine, New York, NY 10021

* Corresponding authors

William J. Netzer, Email: billnetzer@gmail.com, or

Anjana Sinha, Email: sinhaanjana5819@gmail.com, or

Subhash C. Sinha, E-mail: sus2044@med.cornell.edu

Supporting Information (SI)

Contents.

1. Screening Figure of IMTi-1 and 2 analogs and Boc compounds S-1
2. Table S-1. Quantitative structure property relationship (QSPR) analysis S-1
3. HPLC traces for selected compounds S-2
4. NMR spectra of Gleevec isomers and analogs S-10

Fig. S-1. Screening of IMTi-1 and 2 analogs and Boc compounds

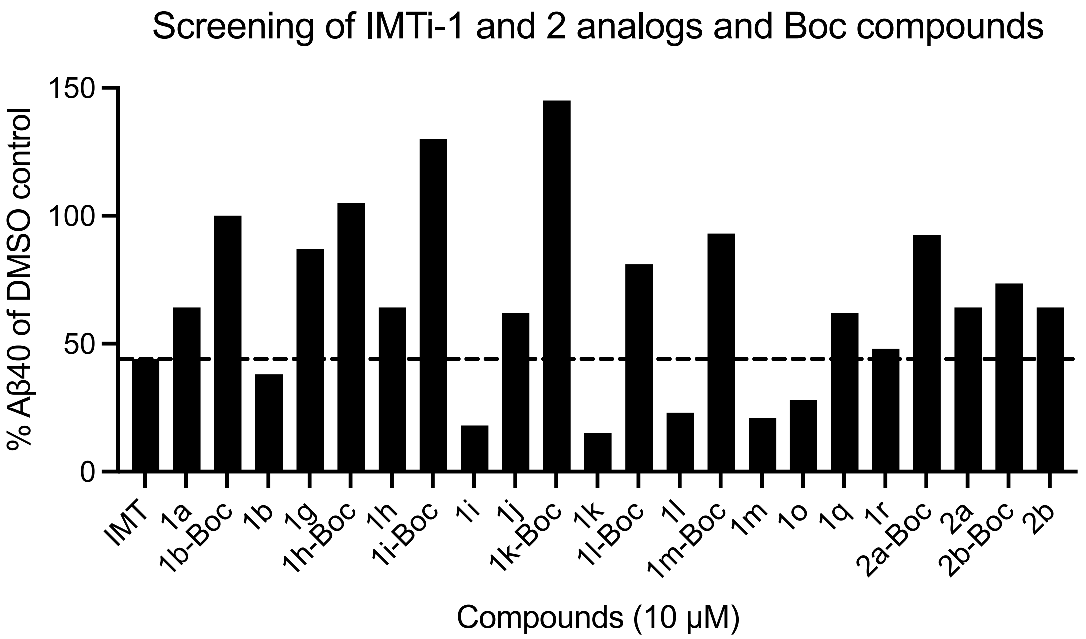

Table S-1. Quantitative structure property relationship (QSPR) analysis to determine structure similarity and the most basic pKa of IMT, IMTi-1 – 3, and their analogs

Comp No	Smiles	FP_sim	Most Basic pKa
IMT	CC1=C(NC2=NC(C3=CC=CN=C3)=CC=N2)C=C(NC(C4=CC=C(CN5CCN(C)CC5)C=C4)=O)C=C1	1	9.02522
IMTi-1	CC1=C(NC2=NC(C3=CN=CC(NC(C4=CC=C(CN5CCN(CC5)C)C=C4)=O)=C3)=CC=N2)C=CC=C1	0.7671	8.96696
IMTi-2	CC1=C(NC(C2=CN=CC=C2)=O)C=C(NC3=NC=CC(C4=CC=C(CN5CCN(C)CC5)C=C4)=N3)C=C1	0.7917	9.16477
IMTi-3	CC(C=CC(CN1CCN(CC2=CC=C(C(NC3=CC=CN=C3)=O)C=C2)CC1)=C4)=C4NC5=NC=CC=N5	0.6667	8.78968
1a	CC1=C(NC2=NC(C3=CC=CC(NC(C4=CC=C(CN5CCN(CC5)C)C=C4)=O)=C3)=CC=N2)C=CC=C1	0.7534	9.01158
1b	CC1=C(NC2=NC(C3=CN=CC(NC(C4=CC=C(CN5CCNCC5)C=C4)=O)=C3)=CC=N2)C=CC=C1	0.6456	9.14014
1c	CC1=C(NC2=NC(C3=CN=CC(NC(C4=CC=C(N5CCN(CC5)C)C=C4)=O)=C3)=CC=N2)C=CC=C1	0.5949	8.72978
1d	CC1=C(NC2=NC(C3=CC=CC(NC(C4=CC=C(N5CCN(CC5)C)C=C4)=O)=C3)=CC=N2)C=CC=C1	0.5823	8.80086
1e	CC1=C(NC2=NC(C3=CN=CC(NC(C4=CC=C(CCN(C)C)C=C4)=O)=C3)=CC=N2)C=CC=C1	0.5244	8.94891
1f	CC1=C(NC2=NC(C3=CC=CC(NC(C4=CC=C(CCN(C)C)C=C4)=O)=C3)=CC=N2)C=CC=C1	0.5122	8.97595
1g	CC1=C(NC2=NC(C3=CN=CC(NC(C4=CC=C(CN(C)C)C=C4)=O)=C3)=CC=N2)C=CC=C1	0.5375	8.53173
1h	CC1=C(NC2=NC(C3=CN=CC(NC(C4=CC=C(CNCC(C)(C)C)C=C4)=O)=C3)=CC=N2)C=CC=C1	0.5119	8.6895
1i	CC1=C(NC2=NC(C3=CC=CC(NC(C4=CC=C(CNCC(C)(C)C)C=C4)=O)=C3)=CC=N2)C=CC=C1	0.5	8.80618
1j	CC1=C(NC2=NC(C3=CN=CC(NC(C4=CC=C(CNC5CCCCC5)C=C4)=O)=C3)=CC=N2)C=CC=C1	0.5176	8.78811
1k	CC1=C(NC2=NC(C3=CC=CC(NC(C4=CC=C(CNC5CCCCC5)C=C4)=O)=C3)=CC=N2)C=CC=C1	0.5059	8.90714
1l	CC1=C(NC2=NC(C3=CN=CC(NC(C4=CC=C(C5CNCCC5)C=C4)=O)=C3)=CC=N2)C=CC=C1	0.4607	9.2167
1m	CC1=C(NC2=NC(C3=CC=CC(NC(C4=CC=C(C5CNCCC5)C=C4)=O)=C3)=CC=N2)C=CC=C1	0.4494	9.24499
1n	CC1=C(NC2=NC(C3=CN=CC(NC(C4=CC=C(C5CN(C)CCC5)C=C4)=O)=C3)=CC=N2)C=CC=C1	0.5172	8.9633
1o	CC1=C(NC2=NC(C3=CC=CC(NC(C4=CC=C(C5CN(C)CCC5)C=C4)=O)=C3)=CC=N2)C=CC=C1	0.5057	9.0332
1p	O=C(C1=CC=C(CCN©C)C=C1)NC2=CC=CC(C3=NC(NC4=CC=CN=C4)=NC=C3)=C2	0.525	8.90998
1q	CC1=CC=CC=C1NC2=NC=CC(C3=CC(C4=CC=C(CN©C)C=C4)=CN=C3)=N2	0.4074	8.7342
2a	O=C(C1=CN=CC=C1)NC2=C(C)C=CC(NC3=NC=CC(C4=CC=C(CNCC(C)(C)C)C=C4)=N3)=C2	0.5301	9.0804
2b	O=C(C1=CN=CC=C1)NC2=C(C)C=CC(NC3=NC=CC(C4=CC=C(CNC5CCCCC5)C=C4)=N3)=C2	0.5357	9.17202

Note: The Extended Connectivity Fingerprint 4 (ECFP4) was generated by RDkit software, and the similarity of compounds to IMT was quantified using the Tanimoto coefficient. The pKa was calculated using Optibrium StarDrop**.** The QSPR analysis was performed by Dr. Han Li at WuXi Apptec, Shanghai, China.

Data File D:\Chemsta...njana\Purify_ABG95_01092019\ABG_95_4_Pure (1) 2019-01-09 13-17-40.D
Sample Name: ABG_95_4_Pure

IMTi-1
≥98% pure

===

Acq. Operator : SYSTEM
Sample Operator : SYSTEM
Acq. Instrument : Prep LC Location : P1-C-09
Injection Date : 1/9/2019 1:18:35 PM Inj : 1
Inj Volume : 50.000 µl
Acq. Method : D:\ChemStation\1\Methods\Anjana\Reverse and Cyclic Gleevec 010819.M
Last changed : 1/8/2019 2:37:40 PM by SYSTEM
Analysis Method : D:\ChemStation\1\Methods\Emily Mui\Reverse and Cyclic Gleevec 010819.M
Last changed : 1/8/2019 2:38:19 PM by SYSTEM
Method Info : Method created 01082019 to run on series of reverse and cyclic gleevec samples at 250 µM in H2o + 0.1% TFA

Sample Info : ABG_95_4 from purified sample

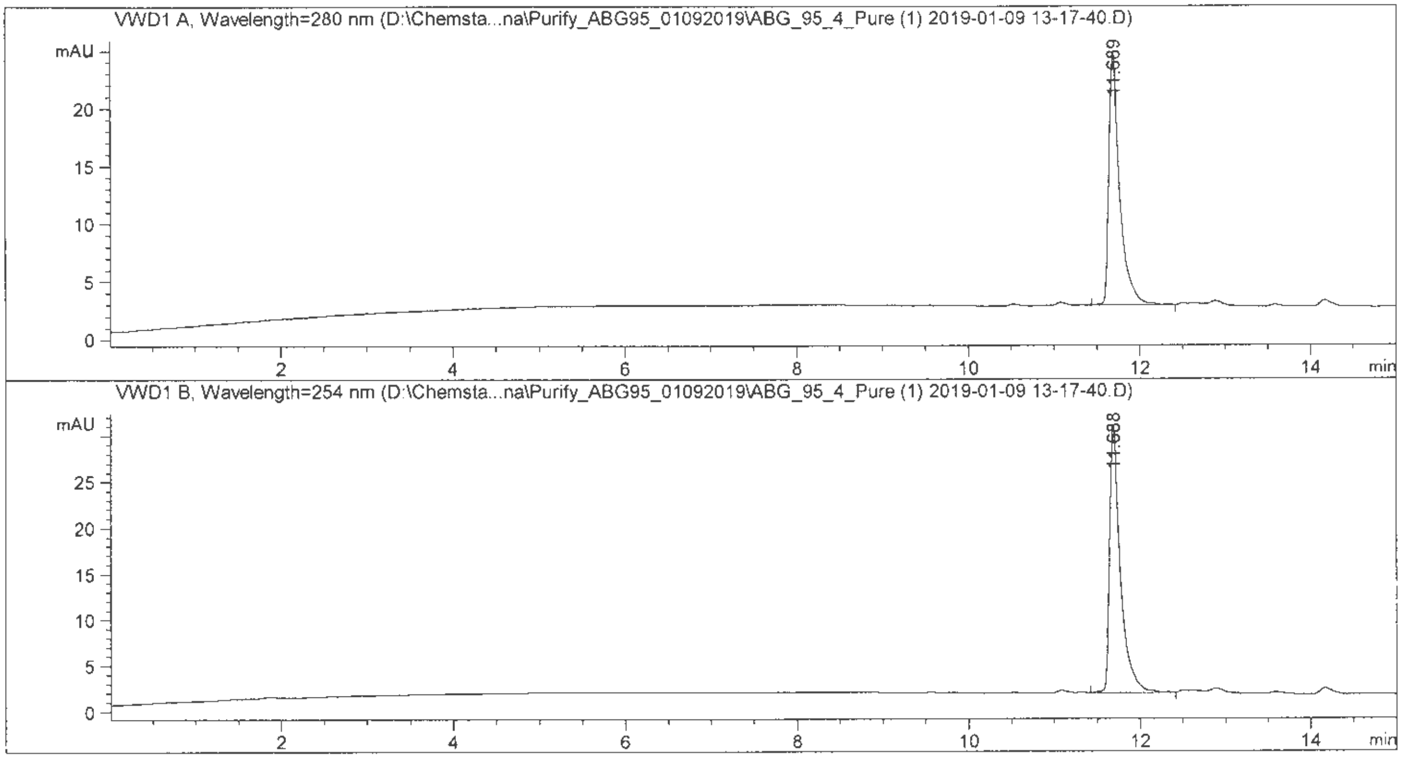

===

Fraction Information

===

No Fractions found.

===

===

===

Area Percent Report

===

Sorted By : Signal
Multiplier : 1.0000
Dilution : 1.0000
Use Multiplier & Dilution Factor with ISTDs

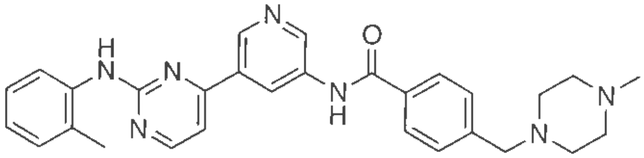

ABG-95

Data File D:\Chemsta...a\Anjana\Purify_ABG256_lPure\ABG_256_Pure (1) 2019-01-09 14-42-29.D
Sample Name: ABG_256_Pure

Comp 1f
>98% pure

==

Acq. Operator	: SYSTEM		
Sample Operator	: SYSTEM		
Acq. Instrument	: Prep LC	Location :	P1-E-08
Injection Date	: 1/9/2019 2:43:30 PM	Inj :	1
		Inj Volume :	50.000 µl
Method	: D:\ChemStation\1\Methods\Anjana\Reverse and Cyclic Gleevec 010819.M		
Last changed	: 1/8/2019 2:37:40 PM by SYSTEM		
Method Info	: Method created 01082019 to run on series of reverse and cyclic gleevec samples at 250 µM prepared in H2O + 0.1% TFA.		

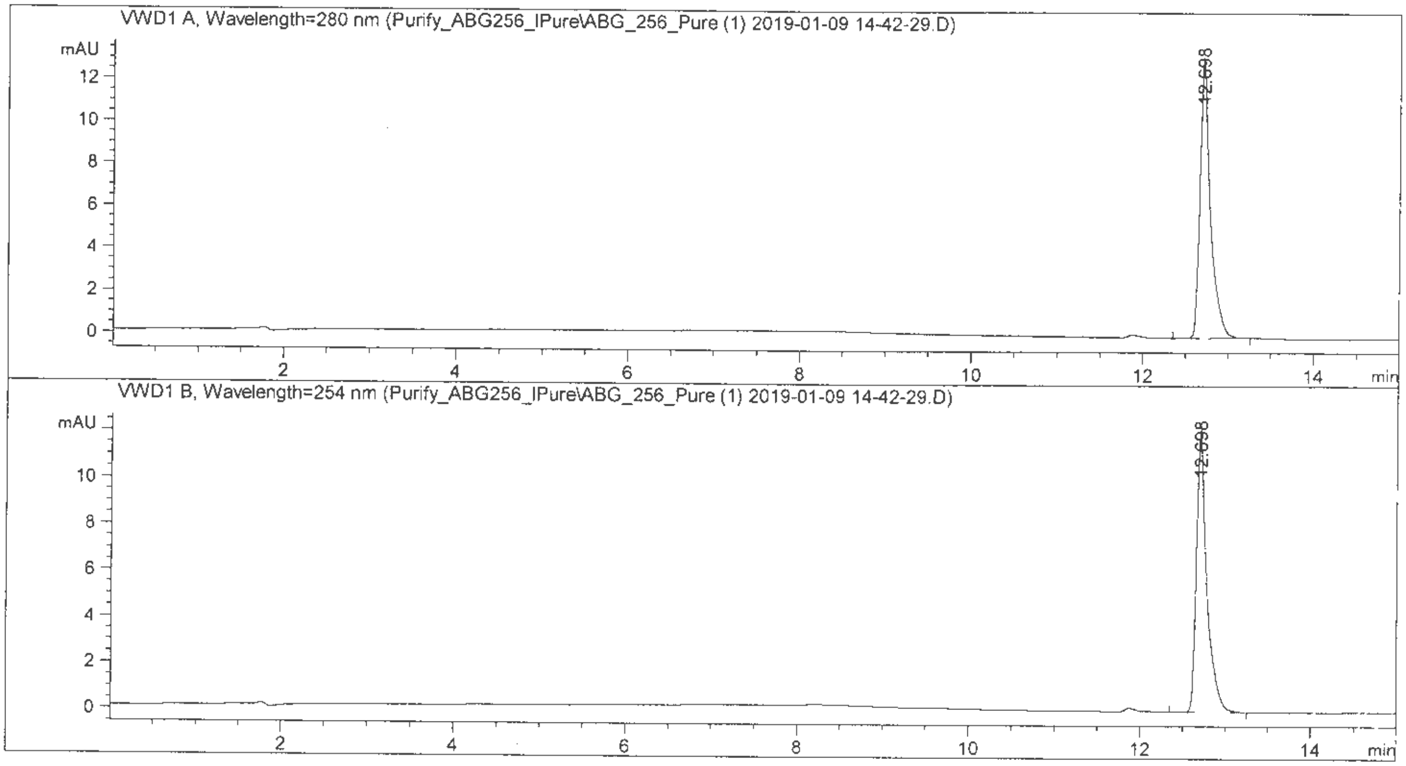

==

Fraction Information

==

No Fractions found.

==

==

==

Area Percent Report

==

Sorted By	:	Signal
Multiplier	:	1.0000
Dilution	:	1.0000

Use Multiplier & Dilution Factor with ISTDs

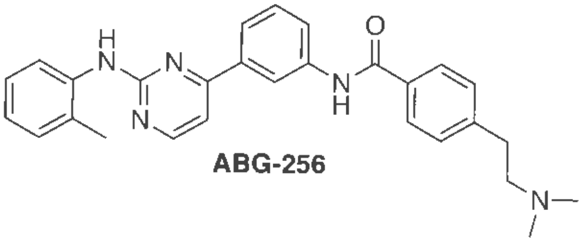

ABG-256

Signal 1: VWD1 A, Wavelength=280 nm

S-3

Data File D:\ChemSta...nd Cyclic Gleevec 01082019 2019-01-08 14-45-36\011-P1-C11-ABG 267.D
Sample Name: ABG 267

Cpd. 1P
>95% pure

===

Acq. Operator	: SYSTEM	Seq. Line :	11
Sample Operator	: SYSTEM		
Acq. Instrument	: Prep LC	Location :	P1-C-11
Injection Date	: 1/8/2019 5:56:56 PM	Inj :	1
		Inj Volume :	50.000 µl

Method : D:\ChemStation\1\Data\Anjana\Gleevec\reverse and Cyclic Gleevec 01082019 2019-01-08 14-45-36\Reverse and Cyclic Gleevec 010819.M (Sequence Method)
Last changed : 1/8/2019 2:37:40 PM by SYSTEM
Method Info : Method created 01082019 to run on series of reverse and cyclic gleevec samples at 250 µM prepared in H2O + 0.1% TFA.

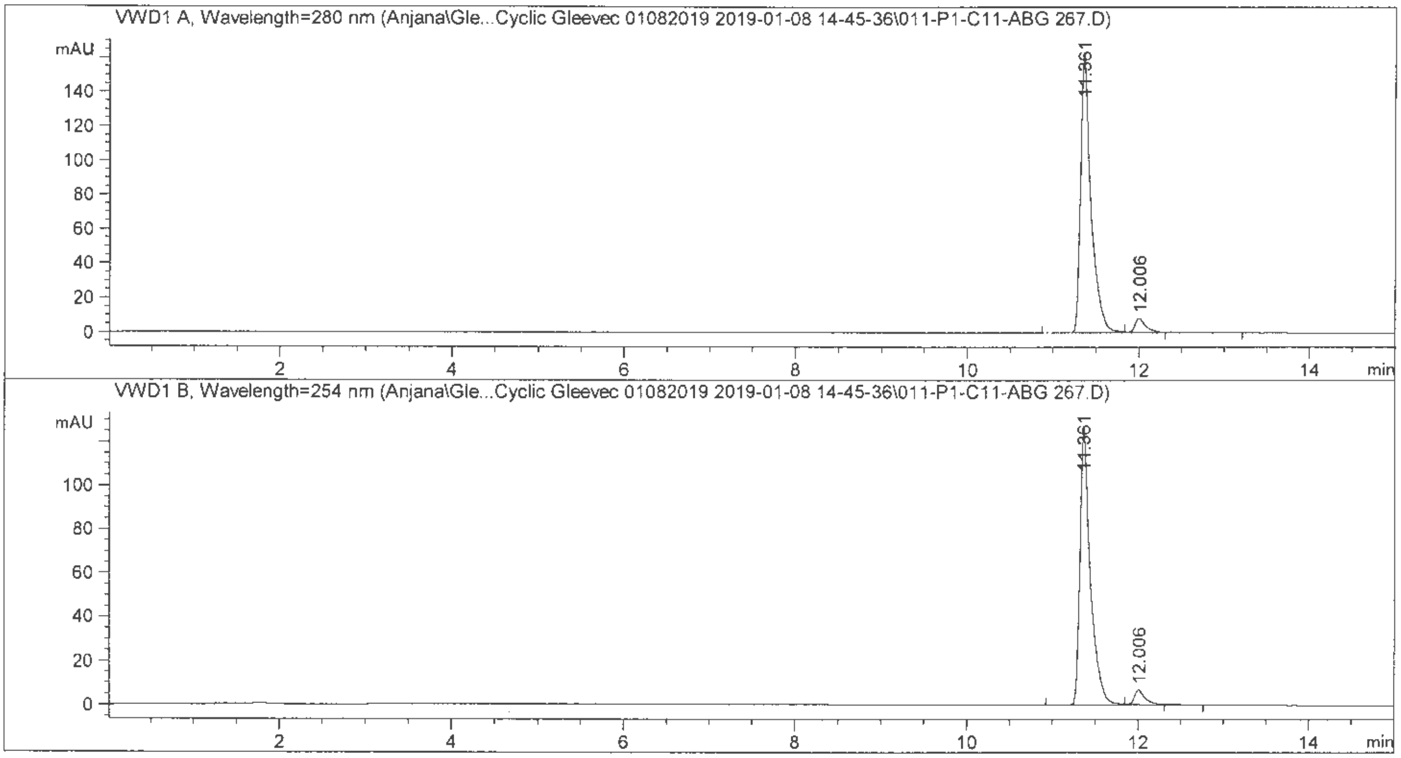

===

Fraction Information

===

No Fractions found.

===
===
===

Area Percent Report

===

Sorted By	:	Signal
Multiplier	:	1.0000
Dilution	:	1.0000

Use Multiplier & Dilution Factor with ISTDs

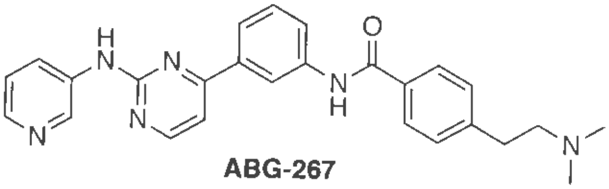

ABG-267

S-4

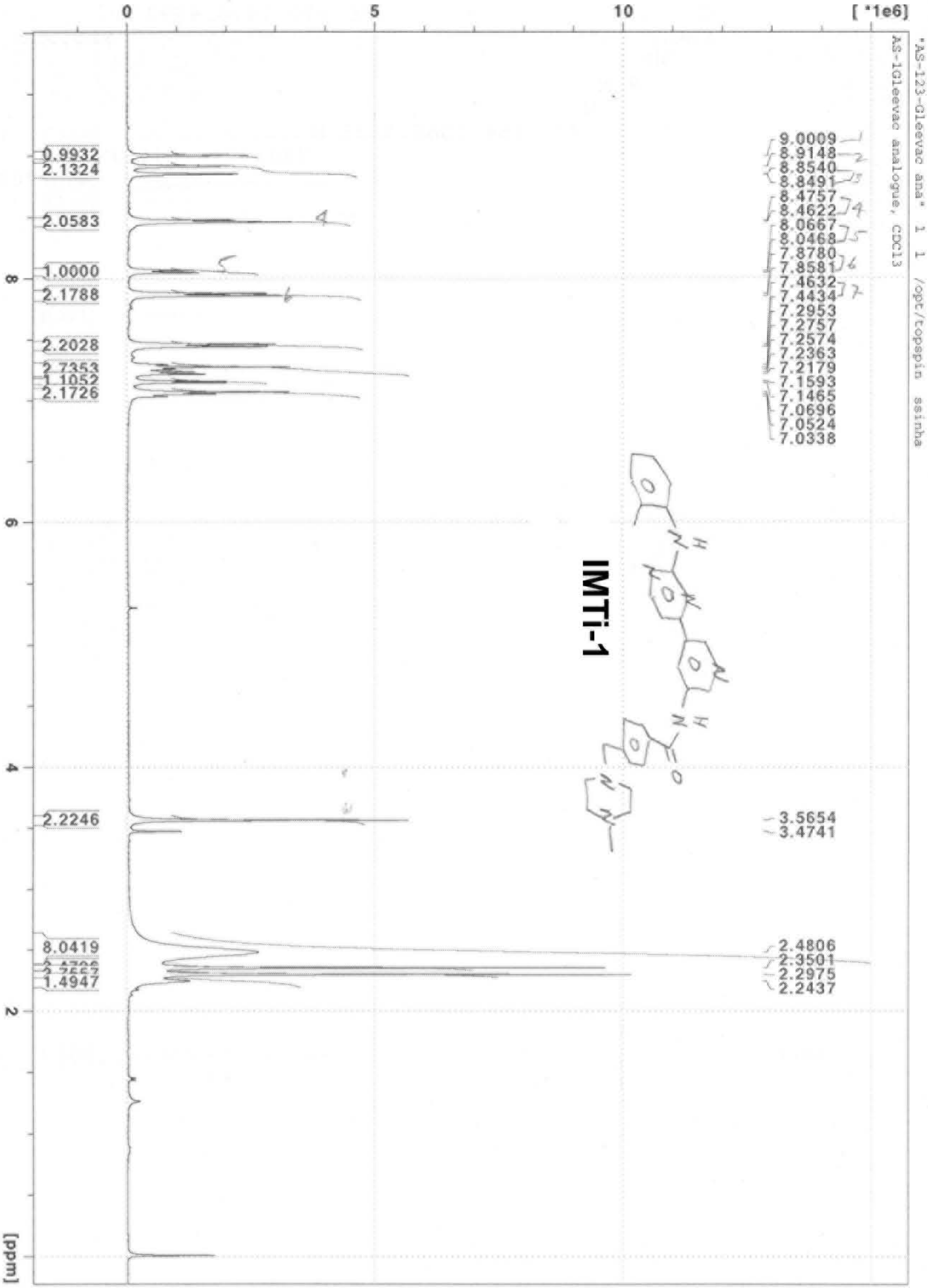
"AS-123-Gleevac ana" 1 1 /opt/topspin ssinha
AS-1Gleevac analogue, CDCl3
[*1e6]
0
5
10
9.0009
8.9148
8.8540
8.8491
8.4757
8.4622
8.0667
8.0468
7.8780
7.8581
7.4632
7.4434
7.2953
7.2757
7.2574
7.2363
7.2179
7.1593
7.1465
7.0696
7.0524
7.0338
3.5654
3.4741
2.4806
2.3501
2.2975
2.2437
0.9932
2.1324
2.0583
1.0000
2.1788
2.2028
2.7353
1.1052
2.1726
2.2246
8.0419
1.4947
8
6
4
2
[ppm]
IMTi-1

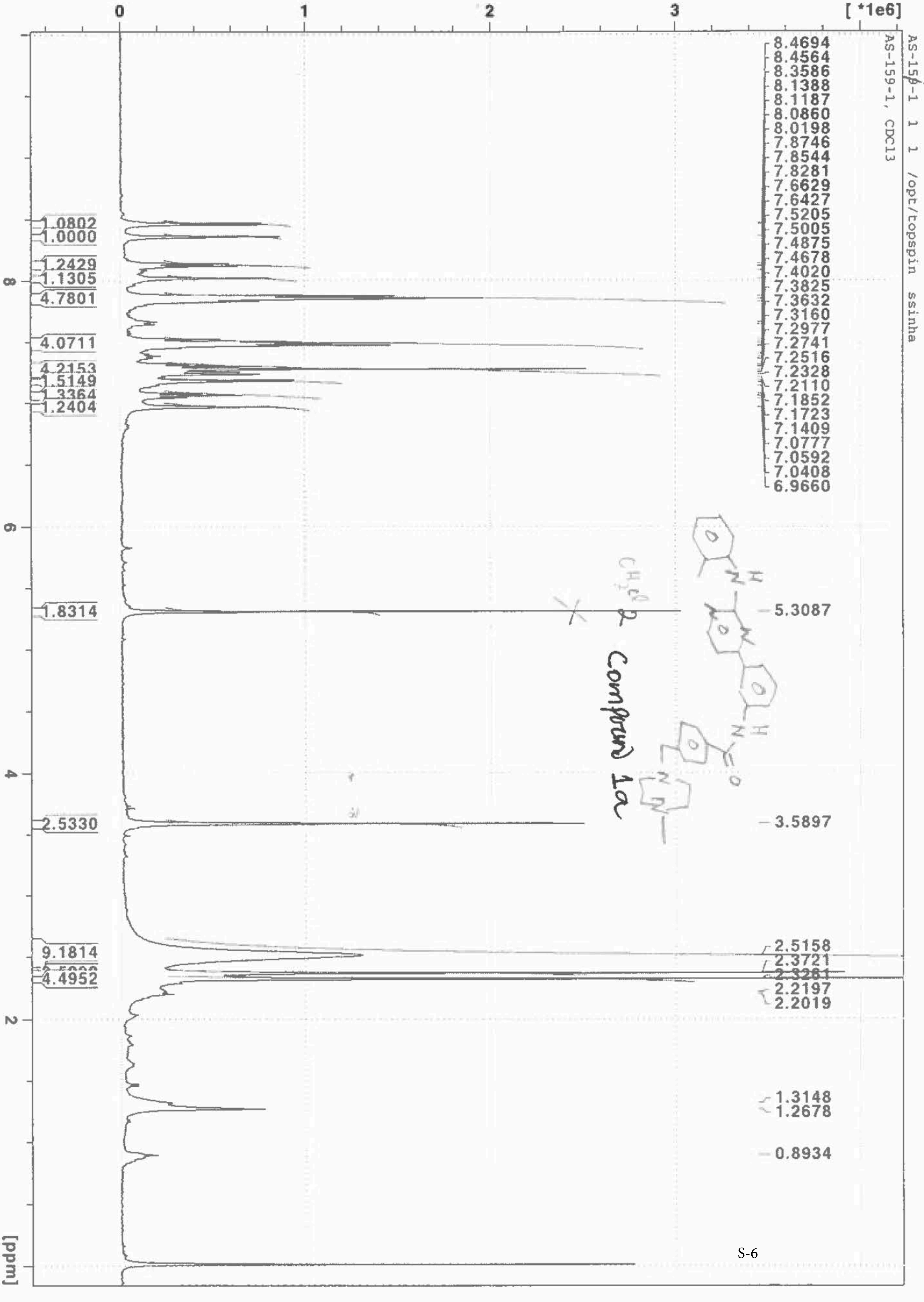
AS-159-1 1 1 /opt/topspin ssinha
AS-159-1, CDCl3
[*1e6]
0
1
2
3
8.4694
8.4564
8.3586
8.1388
8.1187
8.0860
8.0198
7.8746
7.8544
7.8281
7.6629
7.6427
7.5205
7.5005
7.4875
7.4678
7.4020
7.3825
7.3632
7.3160
7.2977
7.2741
7.2516
7.2328
7.2110
7.1852
7.1723
7.1409
7.0777
7.0592
7.0408
6.9660
5.3087
3.5897
2.5158
2.3721
2.3261
2.2197
2.2019
1.3148
1.2678
0.8934
1.0802
1.0000
1.2429
1.1305
4.7801
4.0711
4.2153
1.5149
1.3364
1.2404
1.8314
2.5330
9.1814
4.4952
Compound 1a
[ppm]

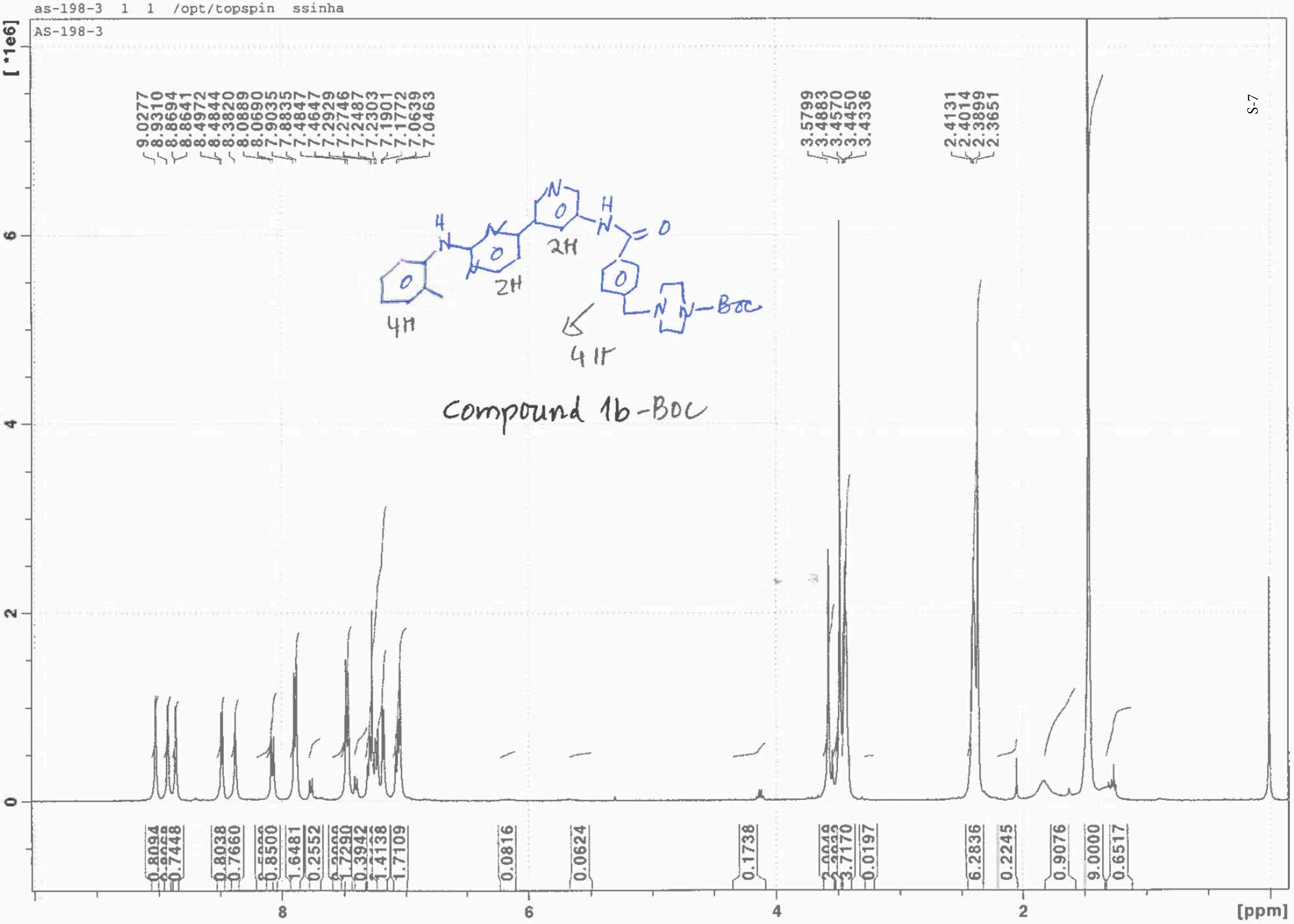
as-198-3 1 1 /opt/topspin ssinha
AS-198-3
[*1e6]
9.0277
8.9310
8.8694
8.8641
8.4972
8.4844
8.3820
8.0889
8.0690
7.9035
7.8835
7.4847
7.4647
7.2929
7.2746
7.2487
7.2303
7.1901
7.1772
7.0639
7.0463
3.5799
3.4883
3.4570
3.4450
3.4336
2.4131
2.4014
2.3899
2.3651
4H
2H
2H
4H
Boc
Compound 1b-Boc
0.8094
0.7448
0.8038
0.7660
0.8500
1.6481
0.2552
1.7290
0.3942
1.4138
1.7109
0.0816
0.0624
0.1738
3.7170
0.0197
6.2836
0.2245
0.9076
9.0000
0.6517
[ppm]

AS-2-91 CDCl3

Cpd. 1c

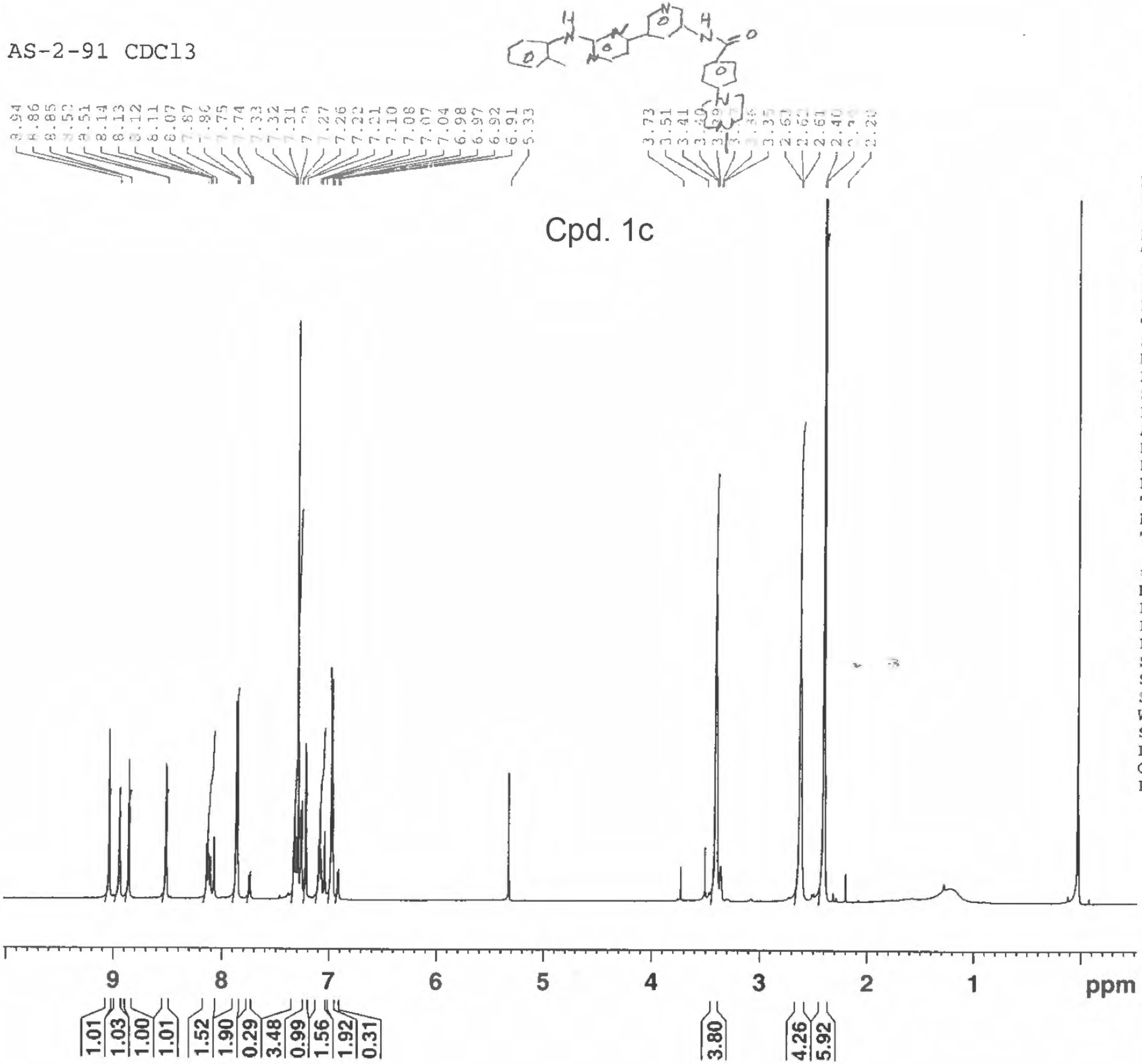

NAME	AS-2-91	
EXPNO	1	
PROCNO	1	
Date_	20160211	
Time	16.44	
INSTRUM	spect	
PROBHD	5 mm CPTCI 1H-	
PULPROG	zg30	
TD	32768	
SOLVENT	CDCl3	
NS	16	
DS	8	
SWH	7788.162	Hz
FIDRES	0.237676	Hz
AQ	2.1038198	sec
RG	28.5	
DW	64.200	usec
DE	6.00	usec
TE	298.1	K
D1	1.00000000	sec
TD0	1	

======== CHANNEL f1 ========

NUC1	1H	
P1	7.45	usec
PL1	4.50	dB
PL1W	5.70400620	W
SFO1	600.1728538	MHz
SI	16384	
SF	600.1699972	MHz
WDW	EM	
SSB	0	
LB	1.00	Hz
GB	0	
PC	1.00	

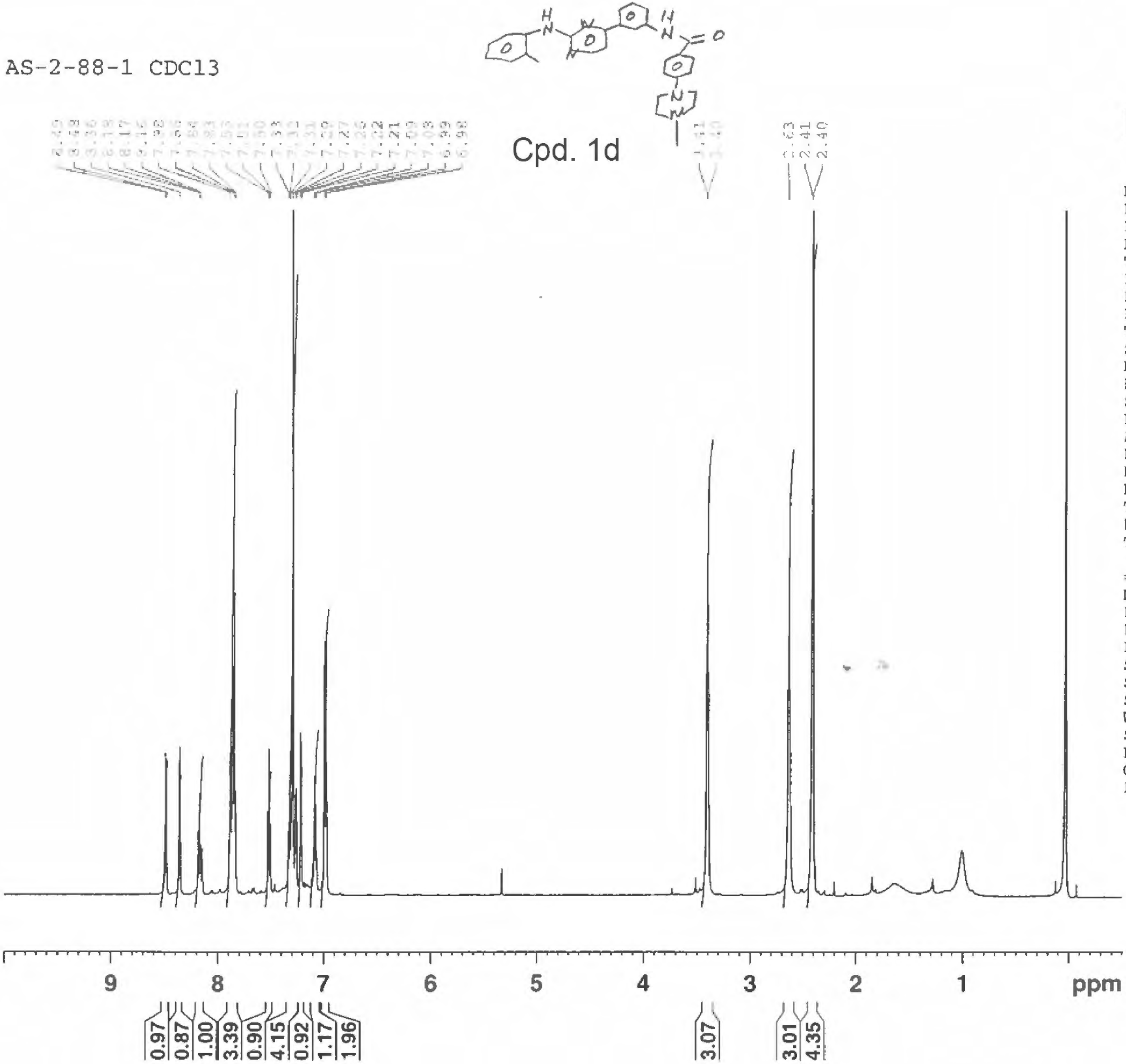

BRUKER

NAME	AS-2-88-1
EXPNO	1
PROCNO	1
Date_	20160211
Time	16.31
INSTRUM	spect
PROBHD	5 mm CPTCI 1H-
PULPROG	zg30
TD	32768
SOLVENT	CDCl3
NS	16
DS	8
SWH	7788.162 Hz
FIDRES	0.237676 Hz
AQ	2.1038198 sec
RG	28.5
DW	64.200 usec
DE	6.00 usec
TE	298.2 K
D1	1.00000000 sec
TD0	1
======== CHANNEL f1 ========	
NUC1	1H
P1	7.45 usec
PL1	4.50 dB
PL1W	5.70400620 W
SFO1	600.1728538 MHz
SI	16384
SF	600.1699972 MHz
WDW	EM
SSB	0
LB	1.00 Hz
GB	0
PC	1.00

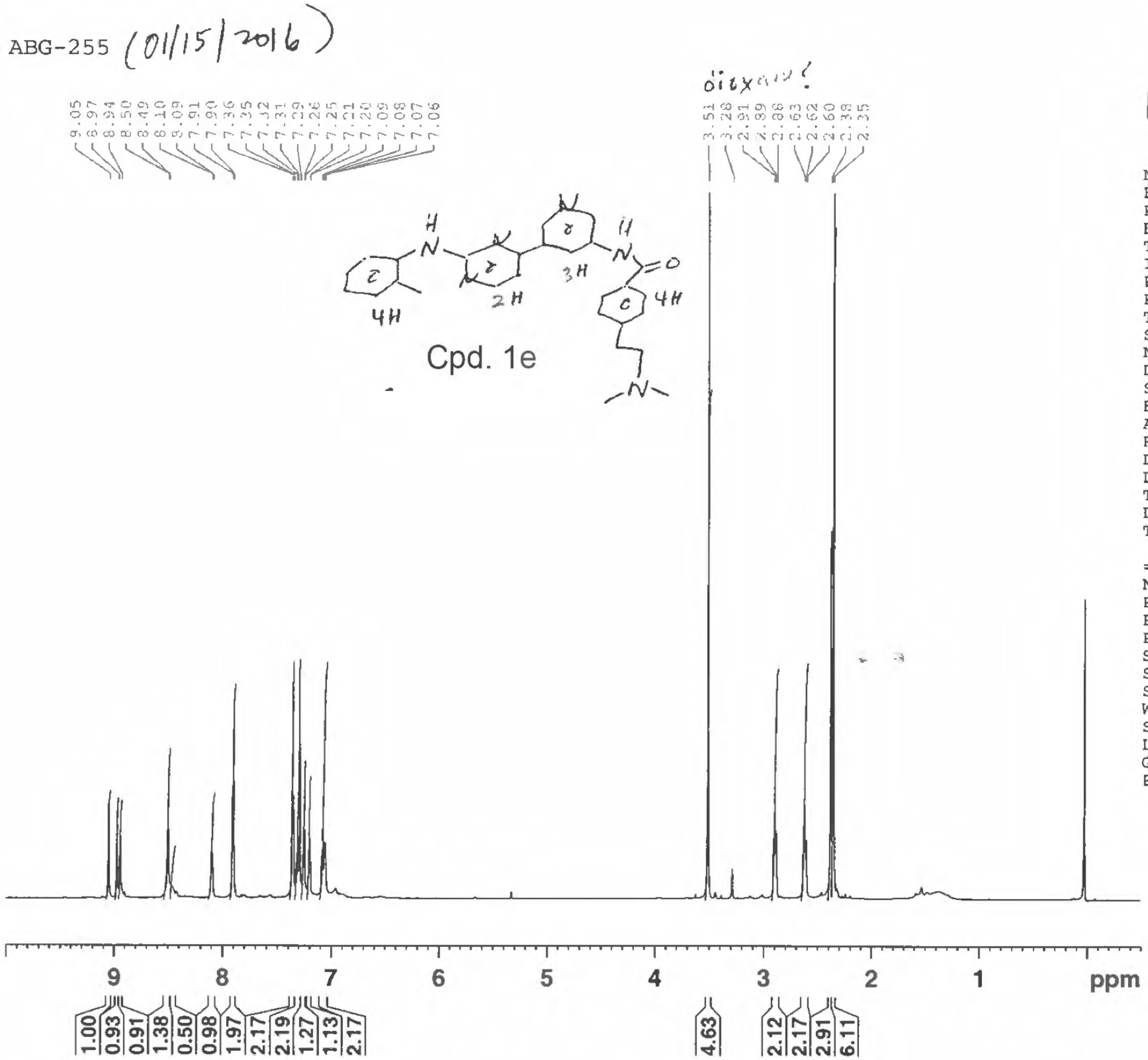

NAME	ABG-255
EXPNO	1
PROCNO	1
Date_	20160121
Time	16.31
INSTRUM	spect
PROBHD	5 mm CPTCI 1H-
PULPROG	zg30
TD	32768
SOLVENT	CDCl3
NS	16
DS	8
SWH	7788.162 Hz
FIDRES	0.237676 Hz
AQ	2.1038198 sec
RG	9
DW	64.200 usec
DE	6.00 usec
TE	298.2 K
D1	1.00000000 sec
TD0	1

======== CHANNEL f1 ========

NUC1	1H
P1	7.45 usec
PL1	4.50 dB
PL1W	5.70400620 W
SFO1	600.1728538 MHz
SI	16384
SF	600.1699972 MHz
WDW	EM
SSB	0
LB	1.00 Hz
GB	0
PC	1.00

ABG-256 R (AS-2-85-L) CDCl3

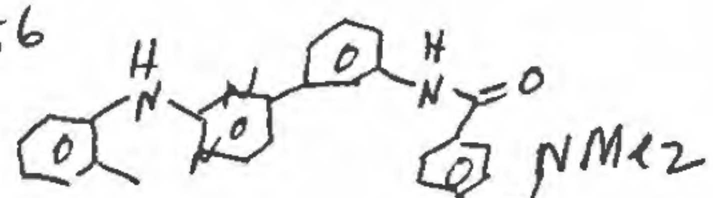

Cpd. 1f

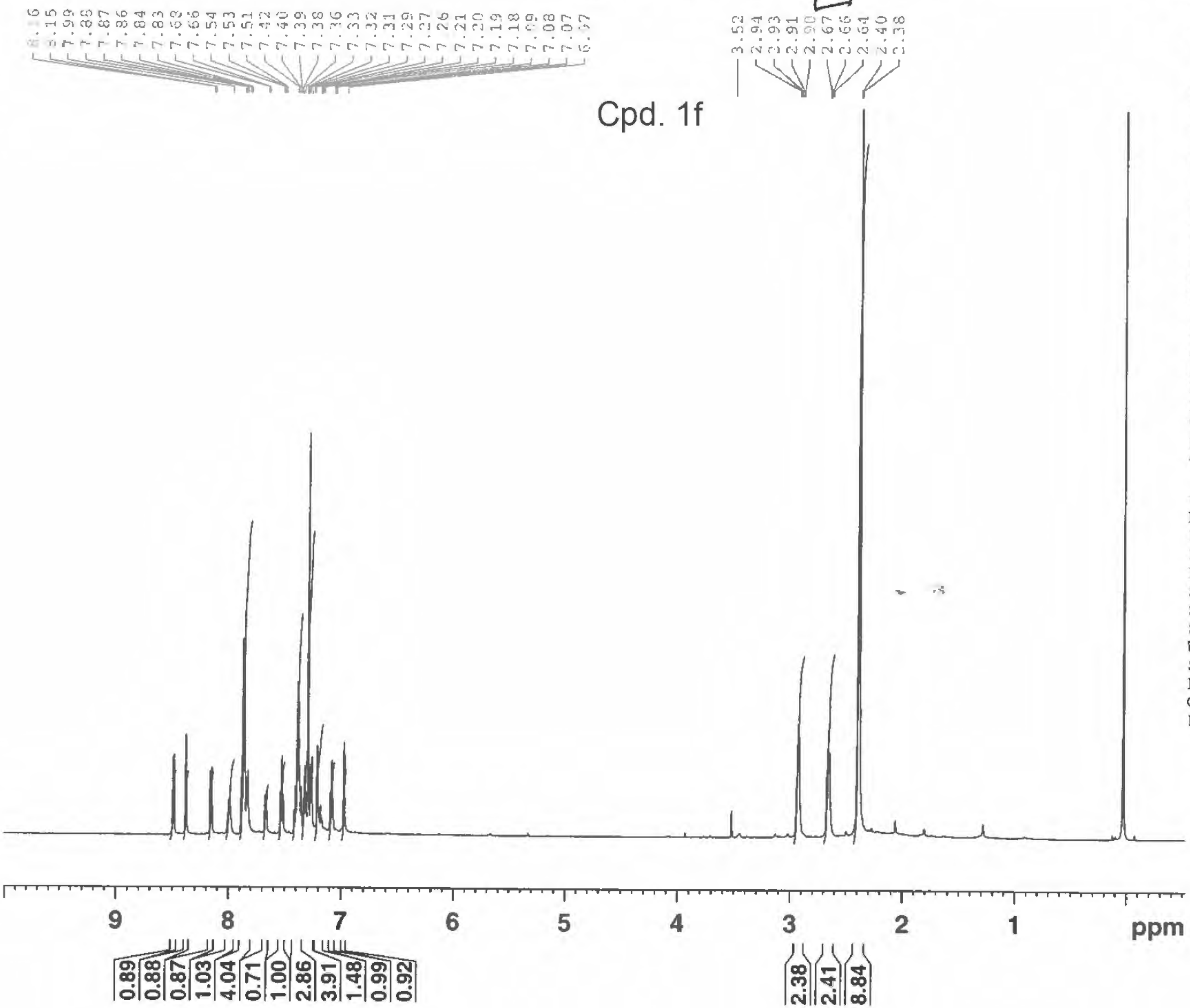

NAME	ABG-256R (As2-85L)
EXPNO	1
PROCNO	1
Date_	20160203
Time	15.26
INSTRUM	spect
PROBHD	5 mm CPTCI 1H-
PULPROG	zg30
TD	32768
SOLVENT	CDCl3
NS	16
DS	8
SWH	7788.162 Hz
FIDRES	0.237676 Hz
AQ	2.1038198 sec
RG	12.7
DW	64.200 usec
DE	6.00 usec
TE	298.2 K
D1	1.00000000 sec
TD0	1
======== CHANNEL f1 ========	
NUC1	1H
P1	7.45 usec
PL1	4.50 dB
PL1W	5.70400620 W
SFO1	600.1728538 MHz
SI	16384
SF	600.1699972 MHz
WDW	EM
SSB	0
LB	1.00 Hz
GB	0
PC	1.00

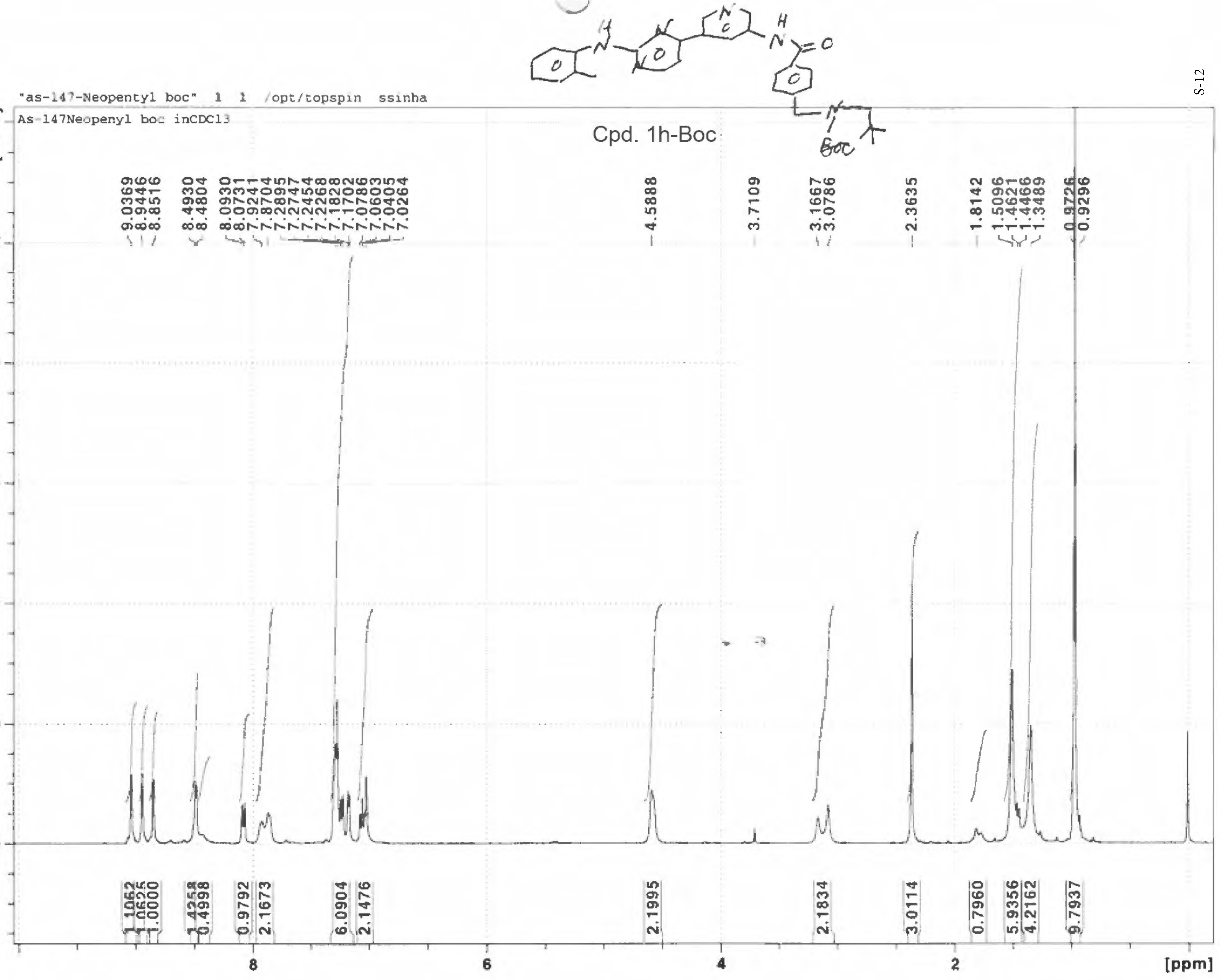
"as-147-Neopentyl boc" 1 1 /opt/topspin ssinha
As-147Neopenyl boc inCDCl3
Cpd. 1h-Boc
[*1e6]
9.0369
8.9446
8.8516
8.4930
8.4804
8.0930
8.0731
7.9241
7.8704
7.2895
7.2747
7.2454
7.2268
7.1828
7.1702
7.0786
7.0603
7.0405
7.0264
4.5888
3.7109
3.1667
3.0786
2.3635
1.8142
1.5096
1.4621
1.4466
1.3489
0.9726
0.9296
1.1062
1.0625
1.0000
1.4258
0.4998
0.9792
2.1673
6.0904
2.1476
2.1995
2.1834
3.0114
0.7960
5.9356
4.2162
9.7937
[ppm]

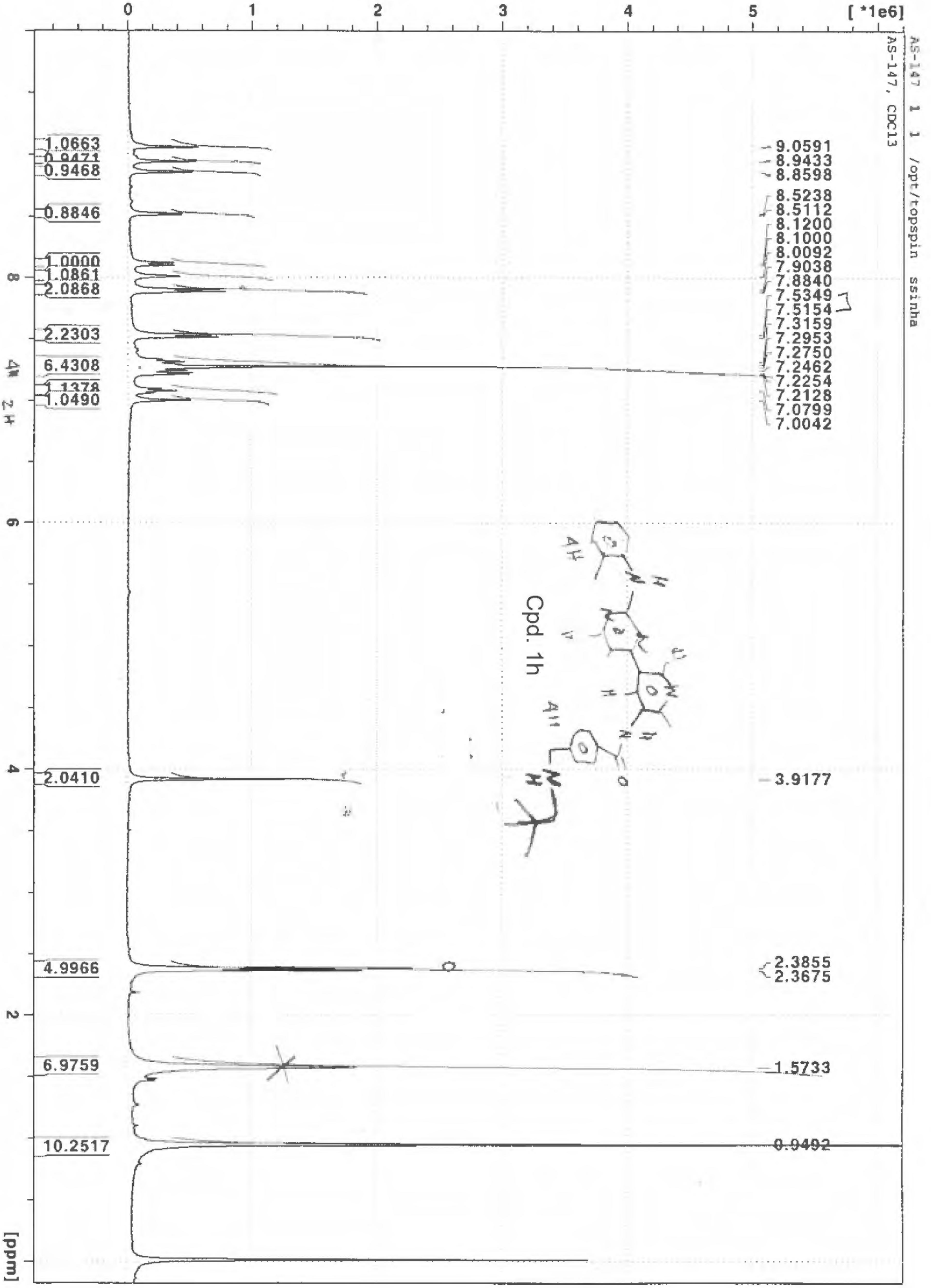
AS-147 1 1 /opt/topspin ssinha
AS-147, CDCl3
Cpd. 1h
9.0591
8.9433
8.8598
8.5238
8.5112
8.1200
8.1000
8.0092
7.9038
7.8840
7.5349
7.5154
7.3159
7.2953
7.2750
7.2462
7.2254
7.2128
7.0799
7.0042
3.9177
2.3855
2.3675
1.5733
0.9492
1.0663
0.9471
0.9468
0.8846
1.0000
1.0861
2.0868
2.2303
6.4308
1.1378
1.0490
2.0410
4.9966
6.9759
10.2517
[*1e6]
[ppm]

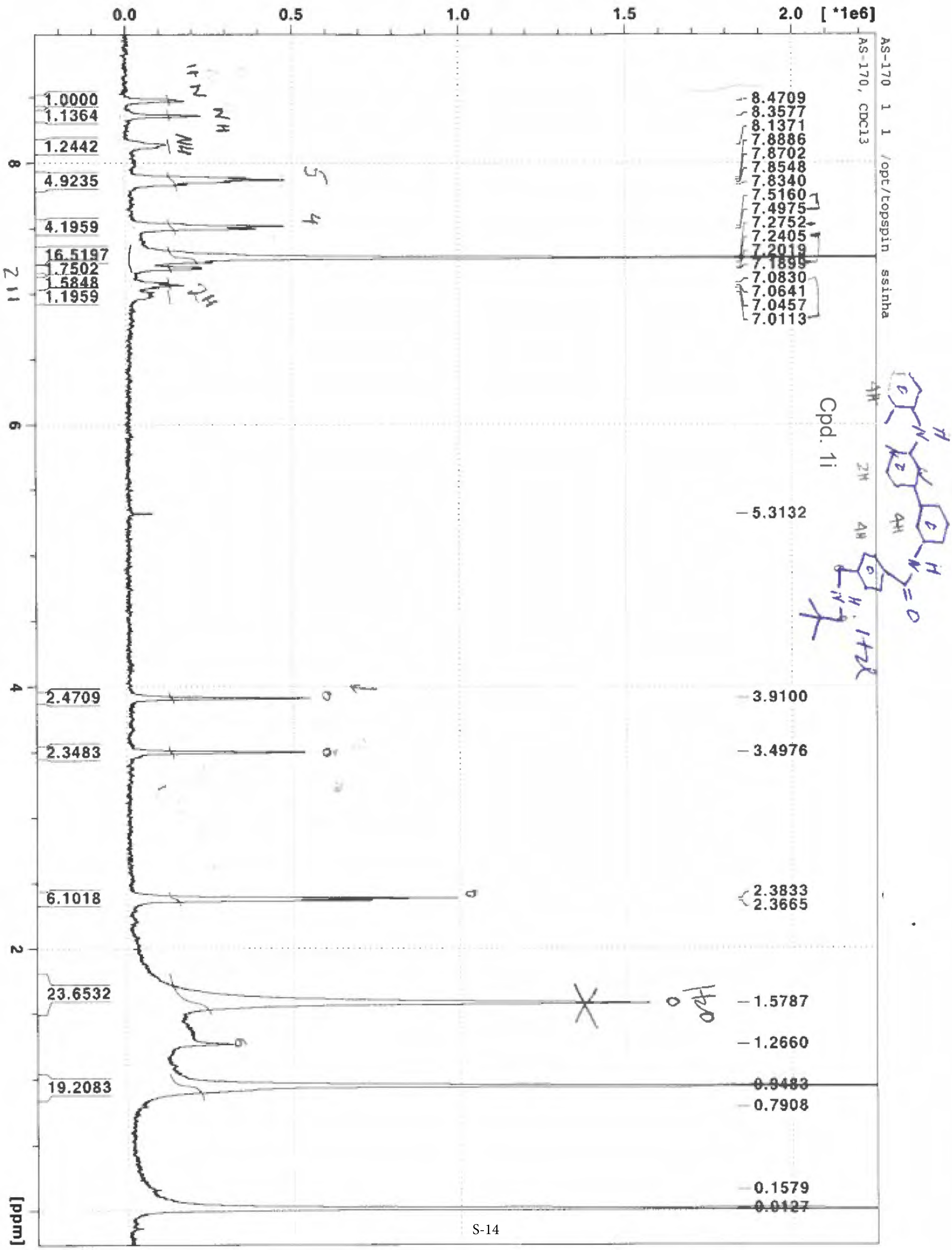

0.0
0.5
1.0
1.5
2.0
[*1e6]
AS-170 1 1 /opt/topspin ssinha
AS-170, CDCl3
Cpd. 1i
[ppm]
8
6
4
2
8.4709
8.3577
8.1371
7.8886
7.8702
7.8548
7.8340
7.5160
7.4975
7.2752
7.2405
7.2019
7.1895
7.0830
7.0641
7.0457
7.0113
5.3132
3.9100
3.4976
2.3833
2.3665
1.5787
1.2660
0.8483
0.7908
0.1579
0.0127
1.0000
1.1364
1.2442
4.9235
4.1959
16.5197
1.7502
1.5848
1.1959
2.4709
2.3483
6.1018
23.6532
19.2083

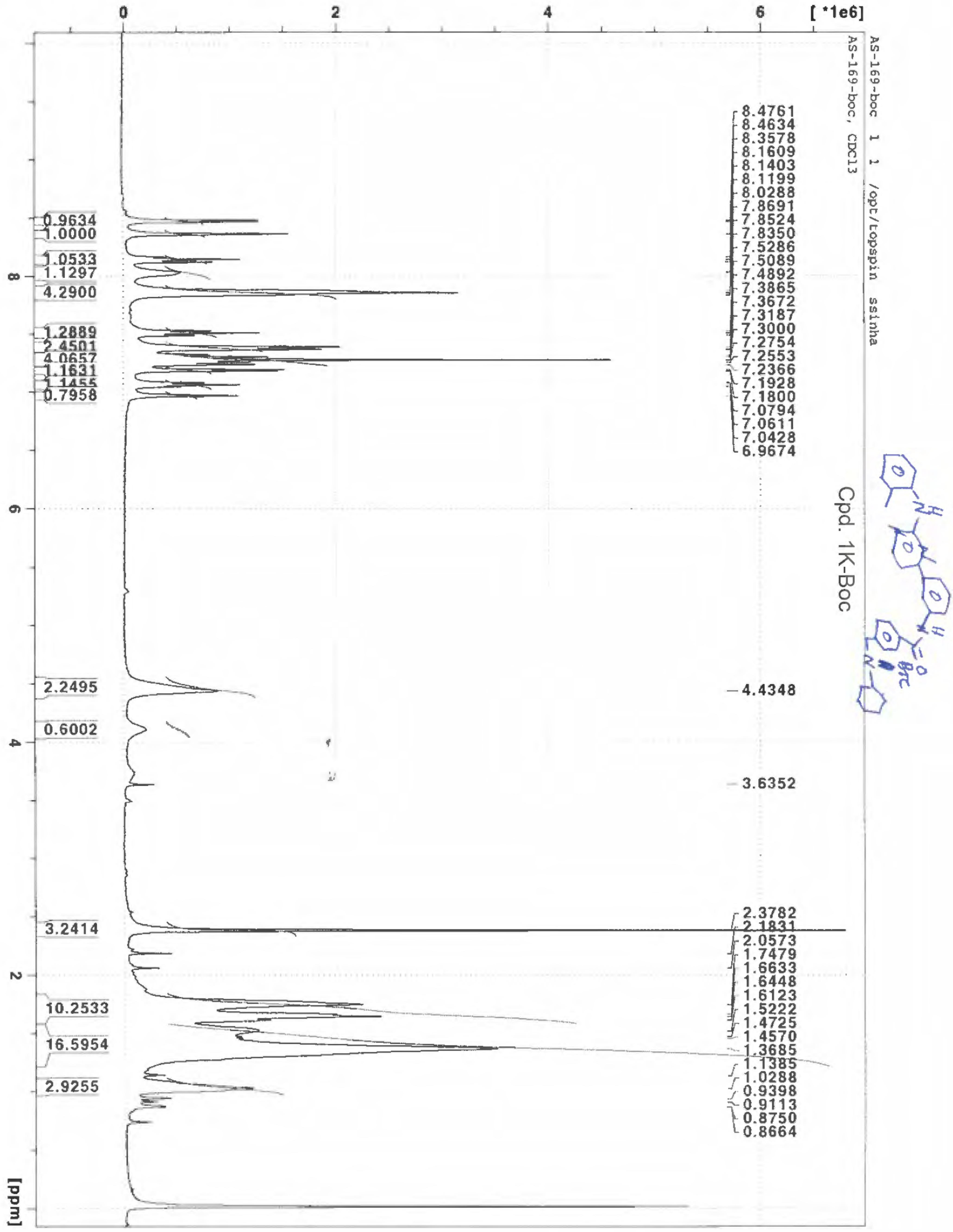

AS-169-boc 1 1 /opt/topspin ssinha
AS-169-boc, CDCl3
Cpd. 1K-Boc
8.4761
8.4634
8.3578
8.1609
8.1403
8.1199
8.0288
7.8691
7.8524
7.8350
7.5286
7.5089
7.4892
7.3865
7.3672
7.3187
7.3000
7.2754
7.2553
7.2366
7.1928
7.1800
7.0794
7.0611
7.0428
6.9674
4.4348
3.6352
2.3782
2.1831
2.0573
1.7479
1.6633
1.6448
1.6123
1.5222
1.4725
1.4570
1.3685
1.1385
1.0288
0.9398
0.9113
0.8750
0.8664
0.9634
1.0000
1.0533
1.1297
4.2900
1.2889
2.4501
4.0657
1.1631
1.1455
0.7958
2.2495
0.6002
3.2414
10.2533
16.5954
2.9255
[ppm]
[*1e6]

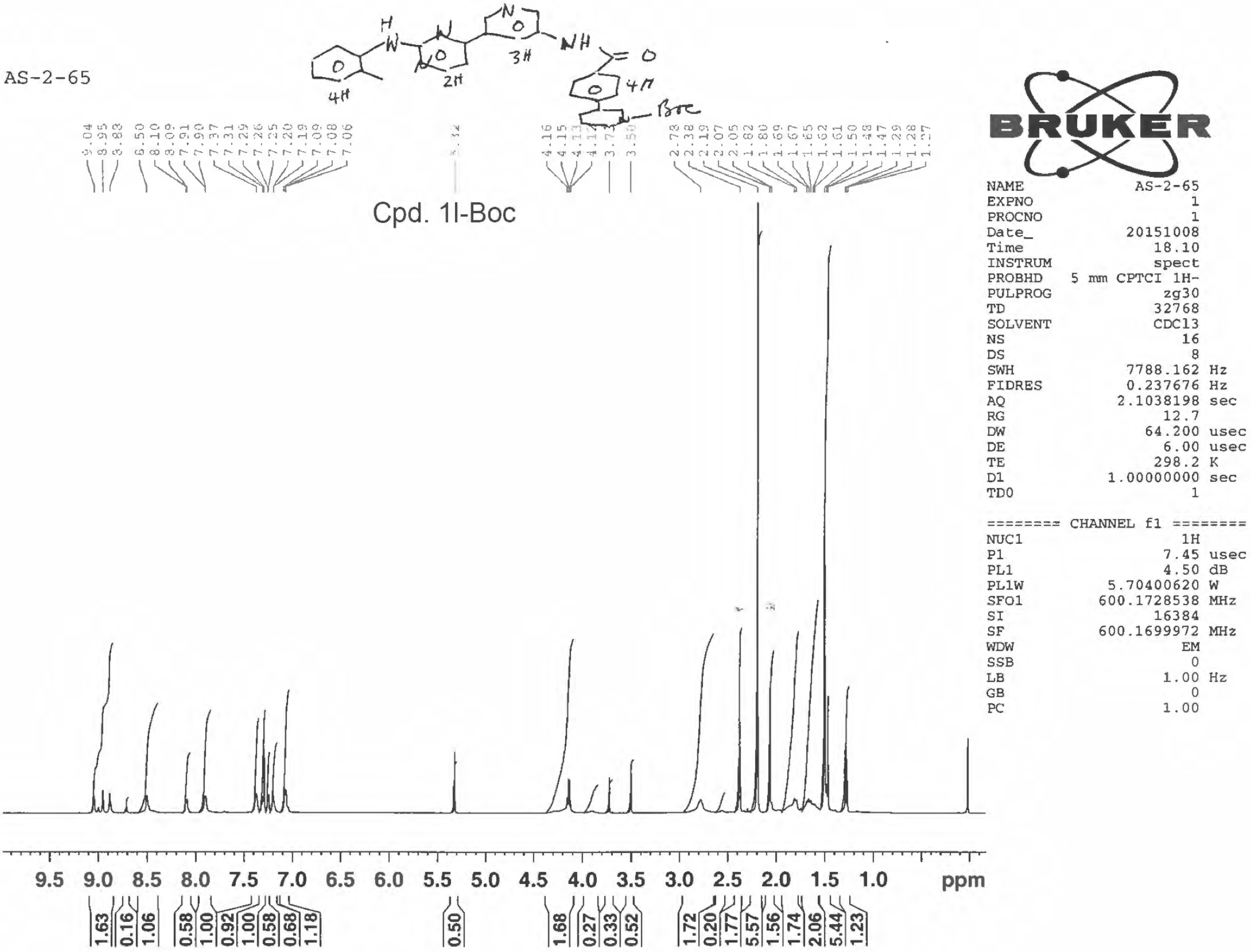
AS-2-65
Cpd. 1l-Boc
4H
2H
3H
4H
Boc
9.04
8.95
8.83
8.50
8.10
8.09
7.91
7.90
7.37
7.31
7.29
7.26
7.25
7.20
7.19
7.09
7.08
7.06
5.32
4.16
4.15
4.13
4.12
3.73
3.50
2.78
2.38
2.19
2.07
2.05
1.82
1.80
1.69
1.67
1.65
1.62
1.61
1.50
1.48
1.47
1.29
1.28
1.27
BRUKER
NAME AS-2-65
EXPNO 1
PROCNO 1
Date_ 20151008
Time 18.10
INSTRUM spect
PROBHD 5 mm CPTCI 1H-
PULPROG zg30
TD 32768
SOLVENT CDCl3
NS 16
DS 8
SWH 7788.162 Hz
FIDRES 0.237676 Hz
AQ 2.1038198 sec
RG 12.7
DW 64.200 usec
DE 6.00 usec
TE 298.2 K
D1 1.00000000 sec
TD0 1
======== CHANNEL f1 ========
NUC1 1H
P1 7.45 usec
PL1 4.50 dB
PL1W 5.70400620 W
SFO1 600.1728538 MHz
SI 16384
SF 600.1699972 MHz
WDW EM
SSB 0
LB 1.00 Hz
GB 0
PC 1.00
9.5 9.0 8.5 8.0 7.5 7.0 6.5 6.0 5.5 5.0 4.5 4.0 3.5 3.0 2.5 2.0 1.5 1.0 ppm
1.63
0.16
1.06
0.58
1.00
0.92
1.00
0.58
0.68
1.18
0.50
1.68
0.27
0.33
0.52
1.72
0.20
1.77
5.57
1.56
1.74
2.06
5.44
1.23

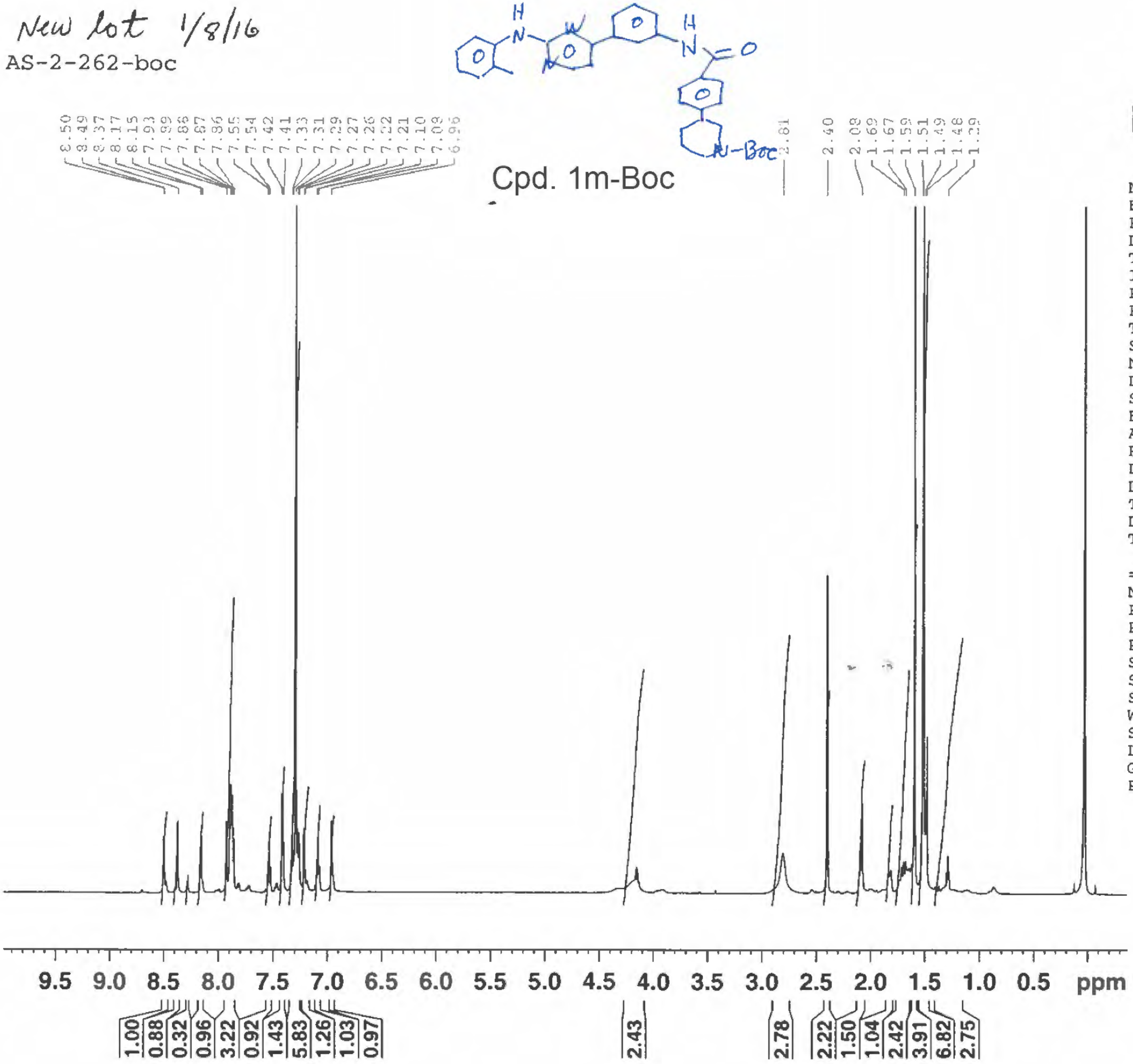

NAME	AS-262-boc
EXPNO	1
PROCNO	1
Date_	20160108
Time	12.06
INSTRUM	spect
PROBHD	5 mm CPTCI 1H-
PULPROG	zg30
TD	32768
SOLVENT	CDCl3
NS	16
DS	8
SWH	7788.162 Hz
FIDRES	0.237676 Hz
AQ	2.1038198 sec
RG	20.2
DW	64.200 usec
DE	6.00 usec
TE	298.2 K
D1	1.00000000 sec
TD0	1
======== CHANNEL f1 ========	
NUC1	1H
P1	7.45 usec
PL1	4.50 dB
PL1W	5.70400620 W
SFO1	600.1728538 MHz
SI	16384
SF	600.1699972 MHz
WDW	EM
SSB	0
LB	1.00 Hz
GB	0
PC	1.00

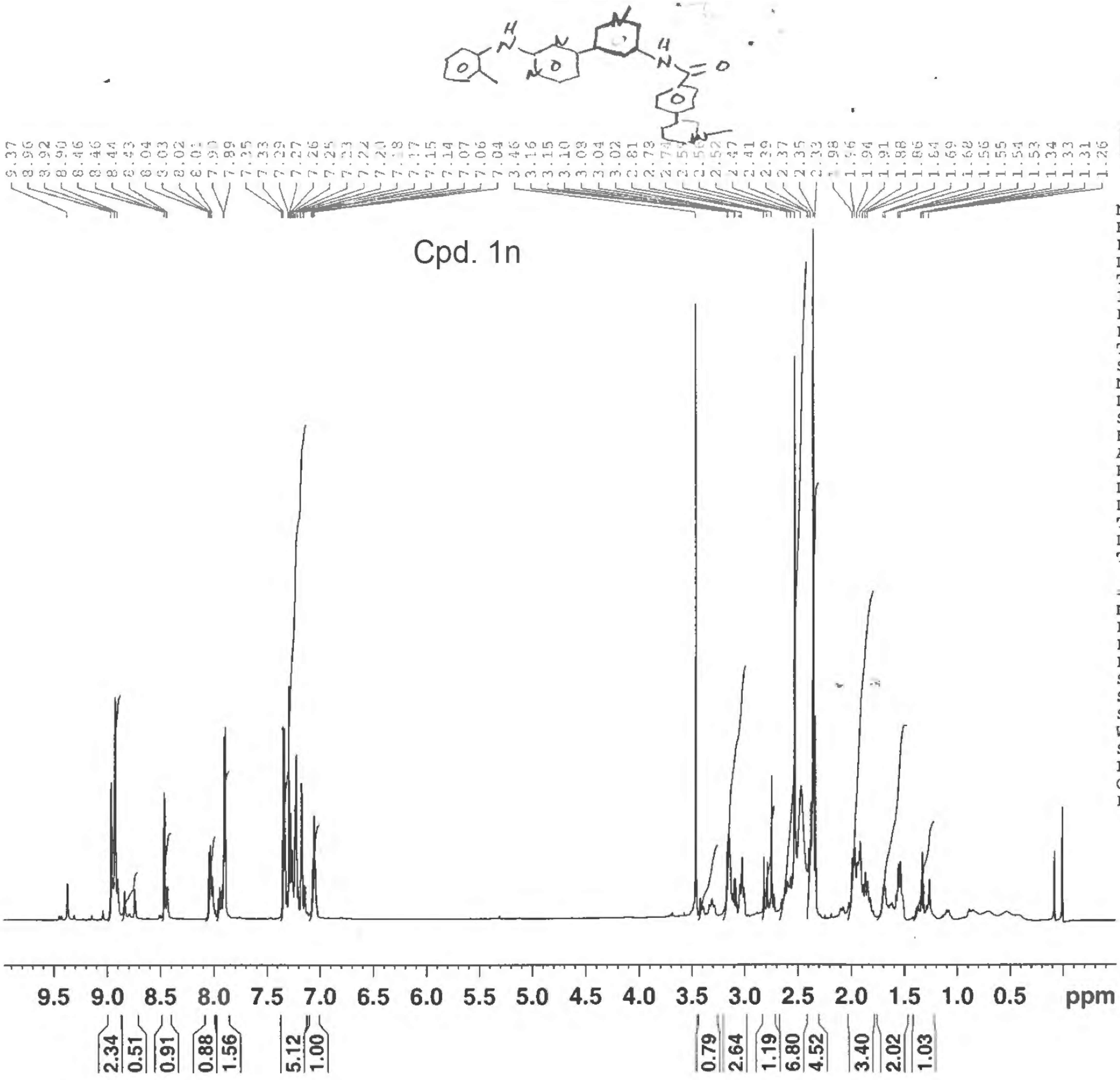

NAME	AS-2-70
EXPNO	1
PROCNO	1
Date_	20151026
Time	16.37
INSTRUM	spect
PROBHD	5 mm CPTCI 1H-
PULPROG	zg30
TD	32768
SOLVENT	CDCl3
NS	16
DS	8
SWH	7788.162 Hz
FIDRES	0.237676 Hz
AQ	2.1038198 sec
RG	12.7
DW	64.200 usec
DE	6.00 usec
TE	298.2 K
D1	1.00000000 sec
TD0	1

======== CHANNEL f1 ========

NUC1	1H
P1	7.45 usec
PL1	4.50 dB
PL1W	5.70400620 W
SFO1	600.1728538 MHz
SI	16384
SF	600.1699972 MHz
WDW	EM
SSB	0
LB	1.00 Hz
GB	0
PC	1.00

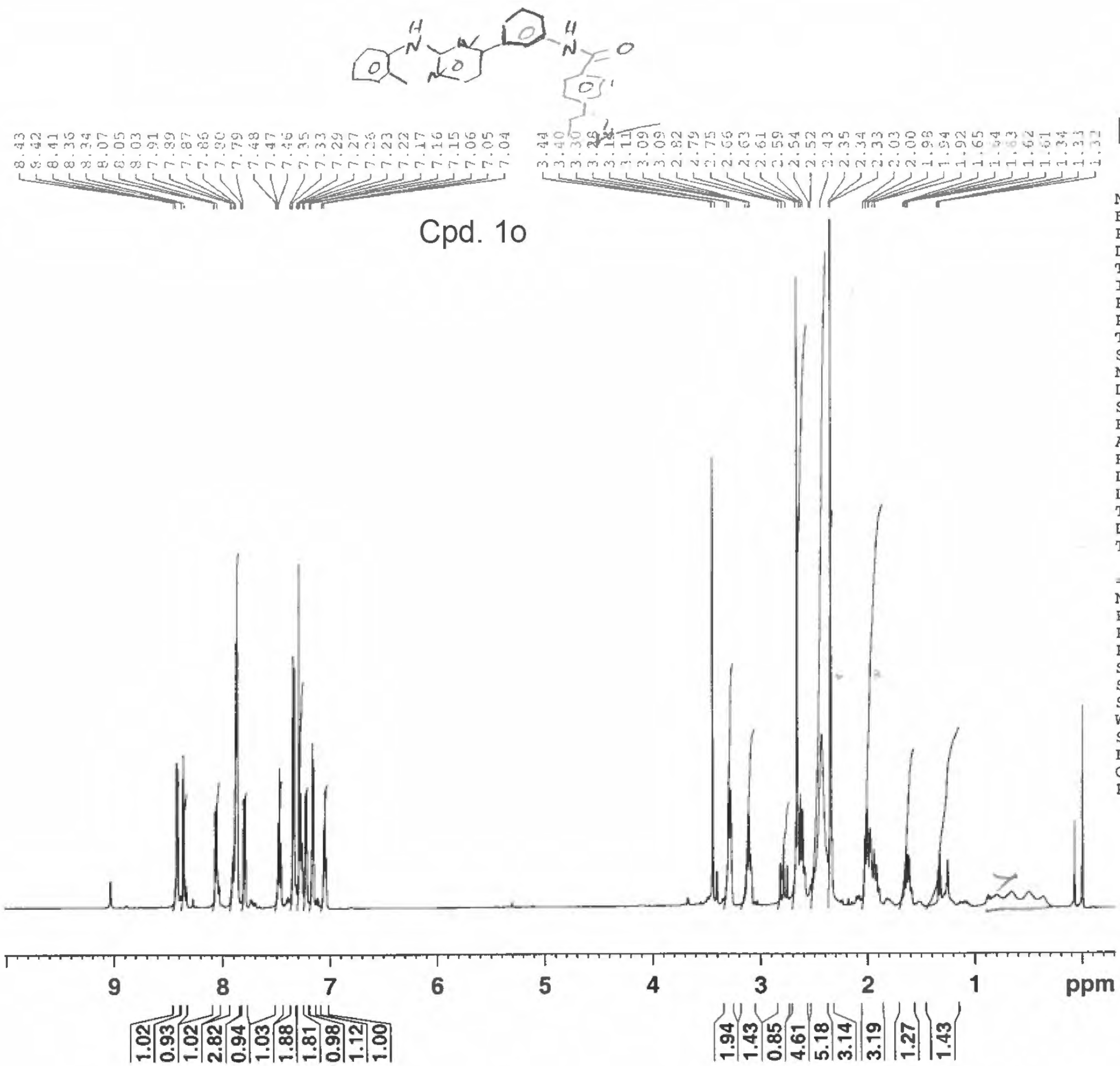

NAME	AS-2-69
EXPNO	1
PROCNO	1
Date_	20151026
Time	16.42
INSTRUM	spect
PROBHD	5 mm CPTCI 1H-
PULPROG	zg30
TD	32768
SOLVENT	CDCl3
NS	16
DS	8
SWH	7788.162 Hz
FIDRES	0.237676 Hz
AQ	2.1038198 sec
RG	12.7
DW	64.200 usec
DE	6.00 usec
TE	298.2 K
D1	1.00000000 sec
TD0	1

======== CHANNEL f1 ========

NUC1	1H
P1	7.45 usec
PL1	4.50 dB
PL1W	5.70400620 W
SFO1	600.1728538 MHz
SI	16384
SF	600.1699972 MHz
WDW	EM
SSB	0
LB	1.00 Hz
GB	0
PC	1.00

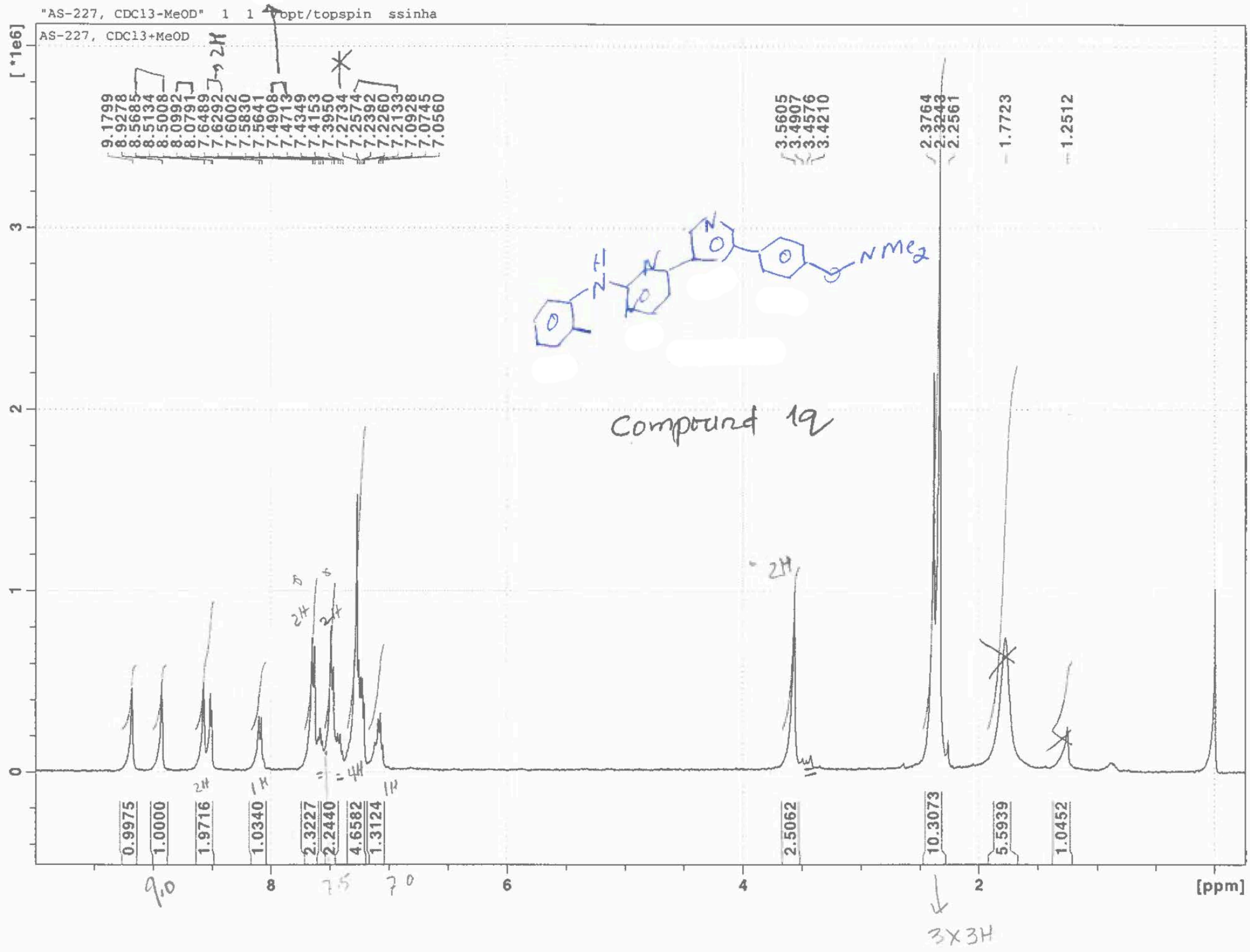

"AS-227, CDCl3-MeOD" 1 1 /opt/topspin ssinha
AS-227, CDCl3+MeOD
[*1e6]
9.1799
8.9278
8.5685
8.5134
8.5008
8.0992
8.0791
7.6489
7.6292
7.6002
7.5830
7.5641
7.4908
7.4713
7.4349
7.4153
7.3950
7.2734
7.2574
7.2392
7.2260
7.2133
7.0928
7.0745
7.0560
3.5605
3.4907
3.4576
3.4210
2.3764
2.3243
2.2561
1.7723
1.2512
Compound 19
NMe2
0.9975
1.0000
1.9716
1.0340
2.3227
2.2440
4.6582
1.3124
2.5062
10.3073
5.5939
1.0452
[ppm]
3X3H

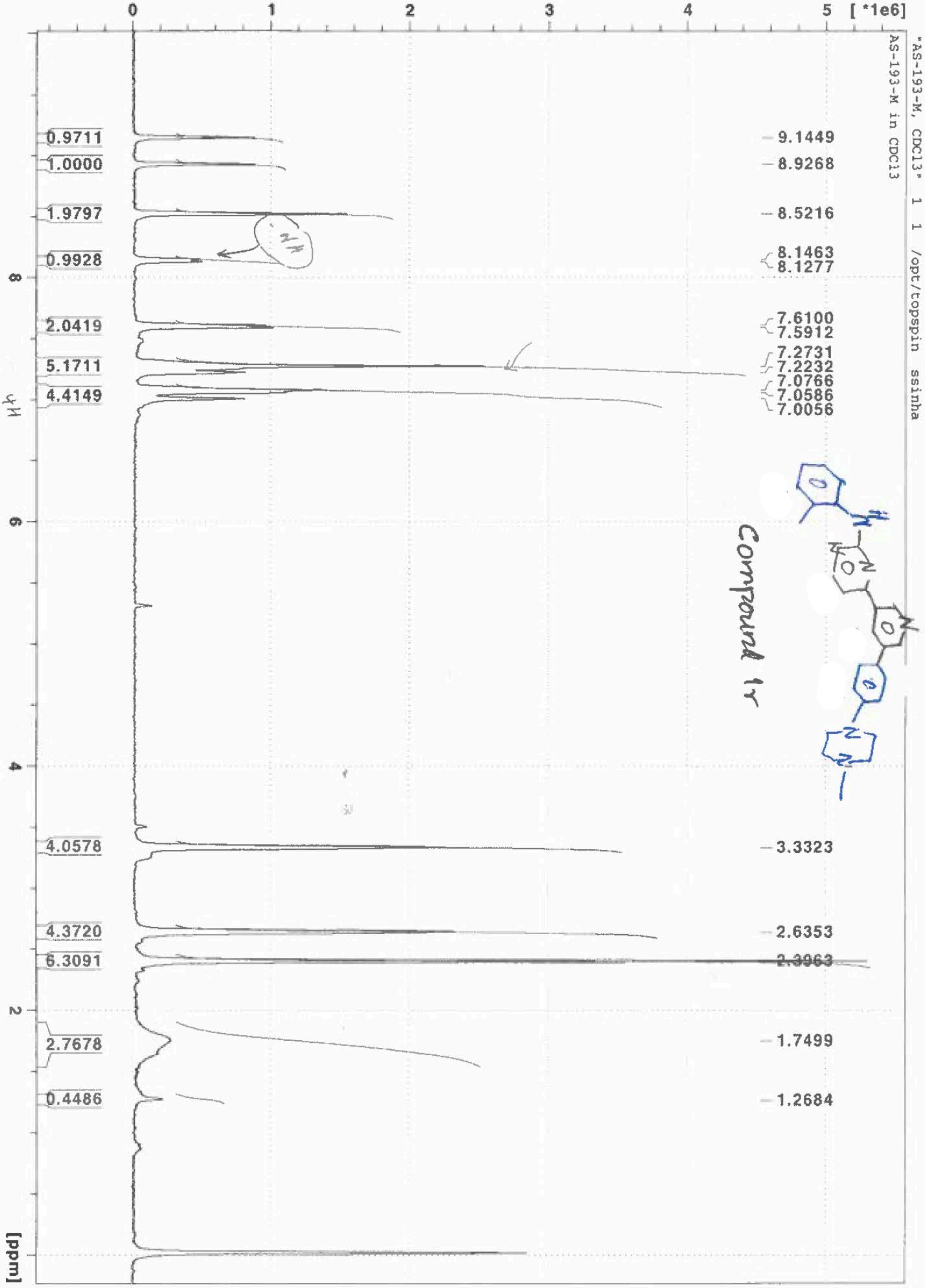
"AS-193-M, CDCl3" 1 1 /opt/topspin ssinha
AS-193-M in CDCl3
[*1e6]
9.1449
8.9268
8.5216
8.1463
8.1277
7.6100
7.5912
7.2731
7.2232
7.0766
7.0586
7.0056
3.3323
2.6353
2.3963
1.7499
1.2684
0.9711
1.0000
1.9797
0.9928
2.0419
5.1711
4.4149
4.0578
4.3720
6.3091
2.7678
0.4486
NH
Compound 1r
[ppm]

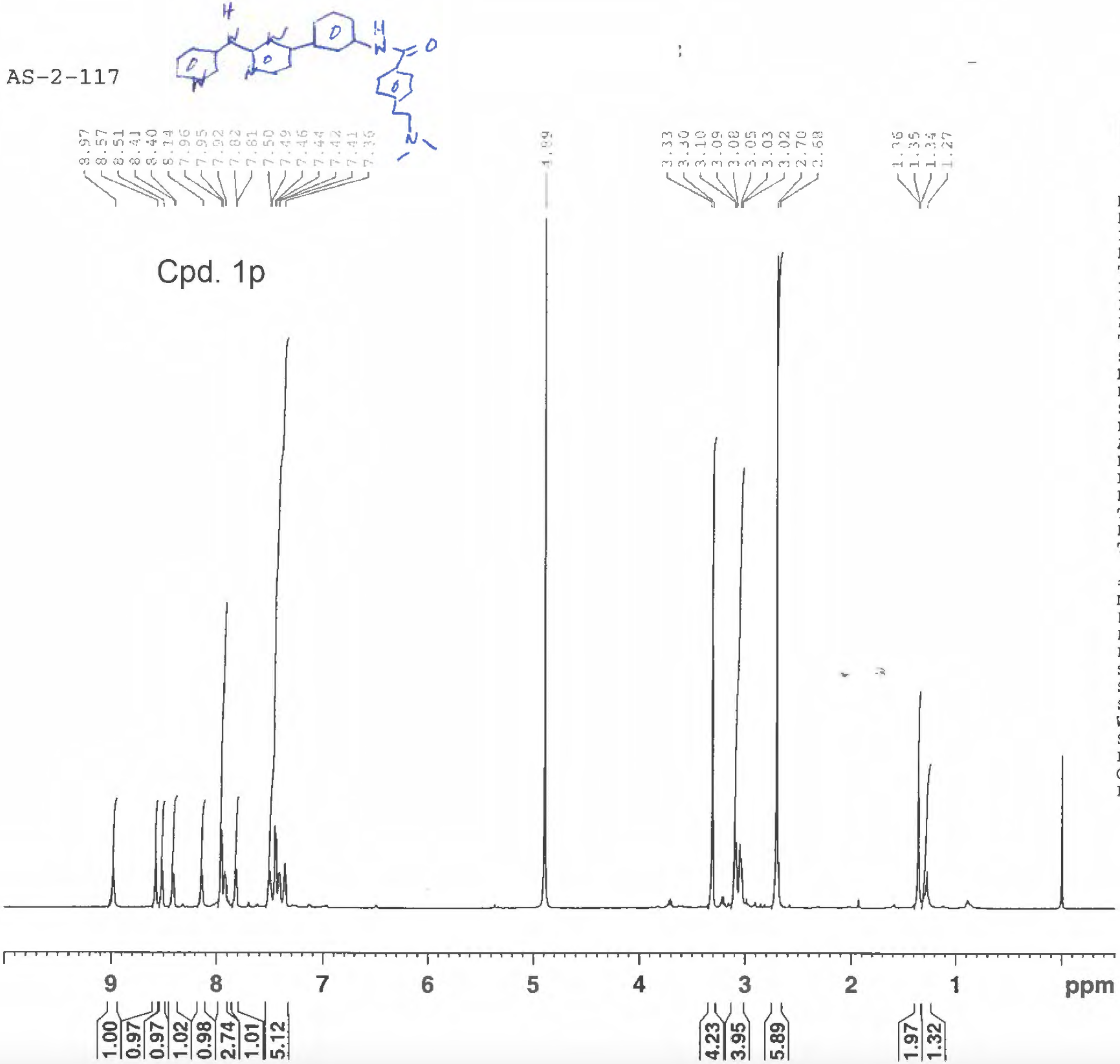

NAME	AS-2-117
EXPNO	1
PROCNO	1
Date_	20160428
Time	16.09
INSTRUM	spect
PROBHD	5 mm CPTCI 1H-
PULPROG	zg30
TD	32768
SOLVENT	MeOD
NS	16
DS	8
SWH	7788.162 Hz
FIDRES	0.237676 Hz
AQ	2.1038198 sec
RG	16
DW	64.200 usec
DE	6.00 usec
TE	298.1 K
D1	1.00000000 sec
TD0	1

======== CHANNEL f1 ========

NUC1	1H
P1	7.45 usec
PL1	4.50 dB
PL1W	5.70400620 W
SFO1	600.1728538 MHz
SI	16384
SF	600.1700169 MHz
WDW	EM
SSB	0
LB	1.00 Hz
GB	0
PC	1.00

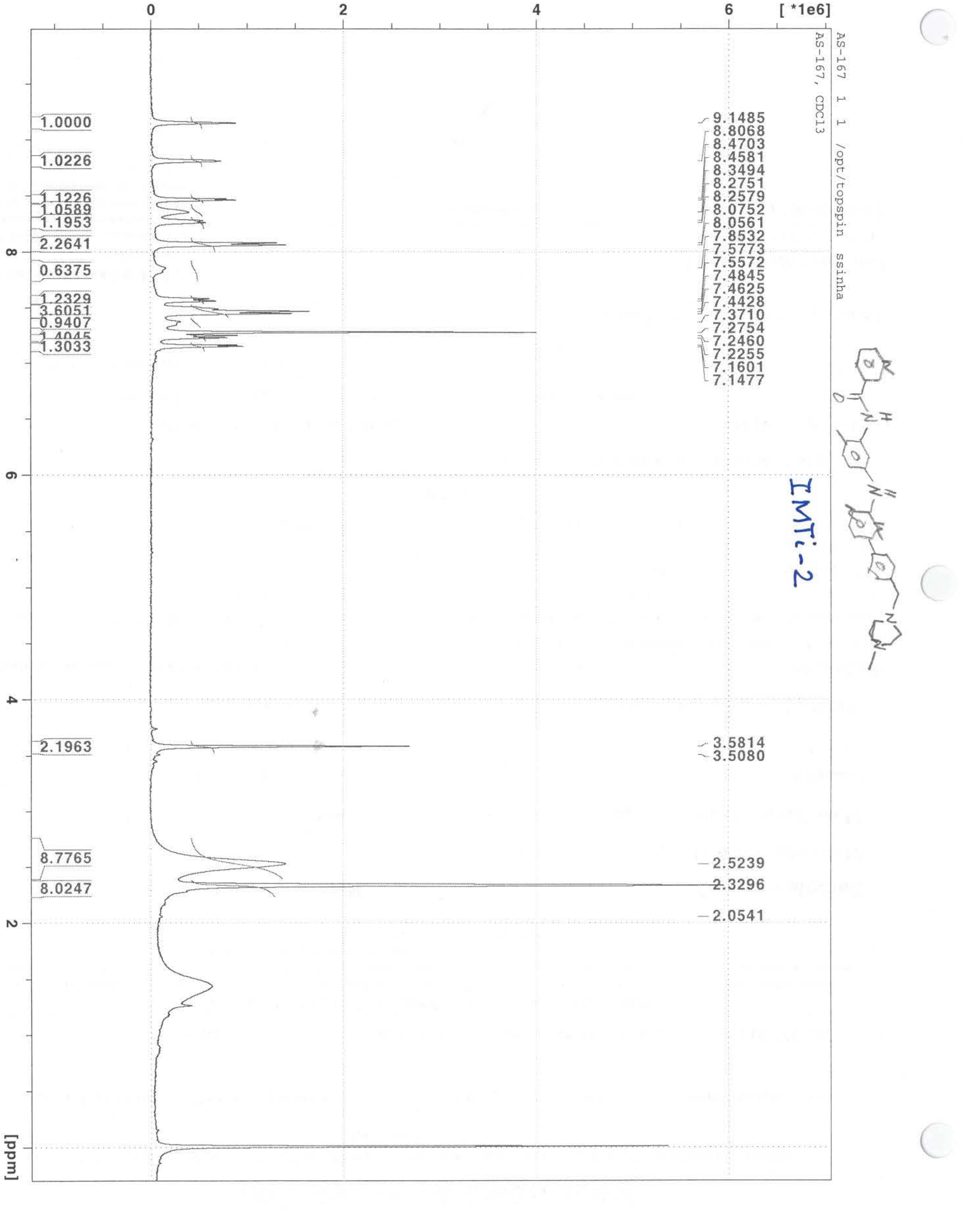
AS-167 1 1 /opt/topspin ssinha
AS-167, CDCl3
[*1e6]
[ppm]
IMTi-2
1.0000
1.0226
1.1226
1.0589
1.1953
2.2641
0.6375
1.2329
3.6051
0.9407
1.4045
1.3033
2.1963
8.7765
8.0247
9.1485
8.8068
8.4703
8.4581
8.3494
8.2751
8.2579
8.0752
8.0561
7.8532
7.5773
7.5572
7.4845
7.4625
7.4428
7.3710
7.2754
7.2460
7.2255
7.1601
7.1477
3.5814
3.5080
2.5239
2.3296
2.0541

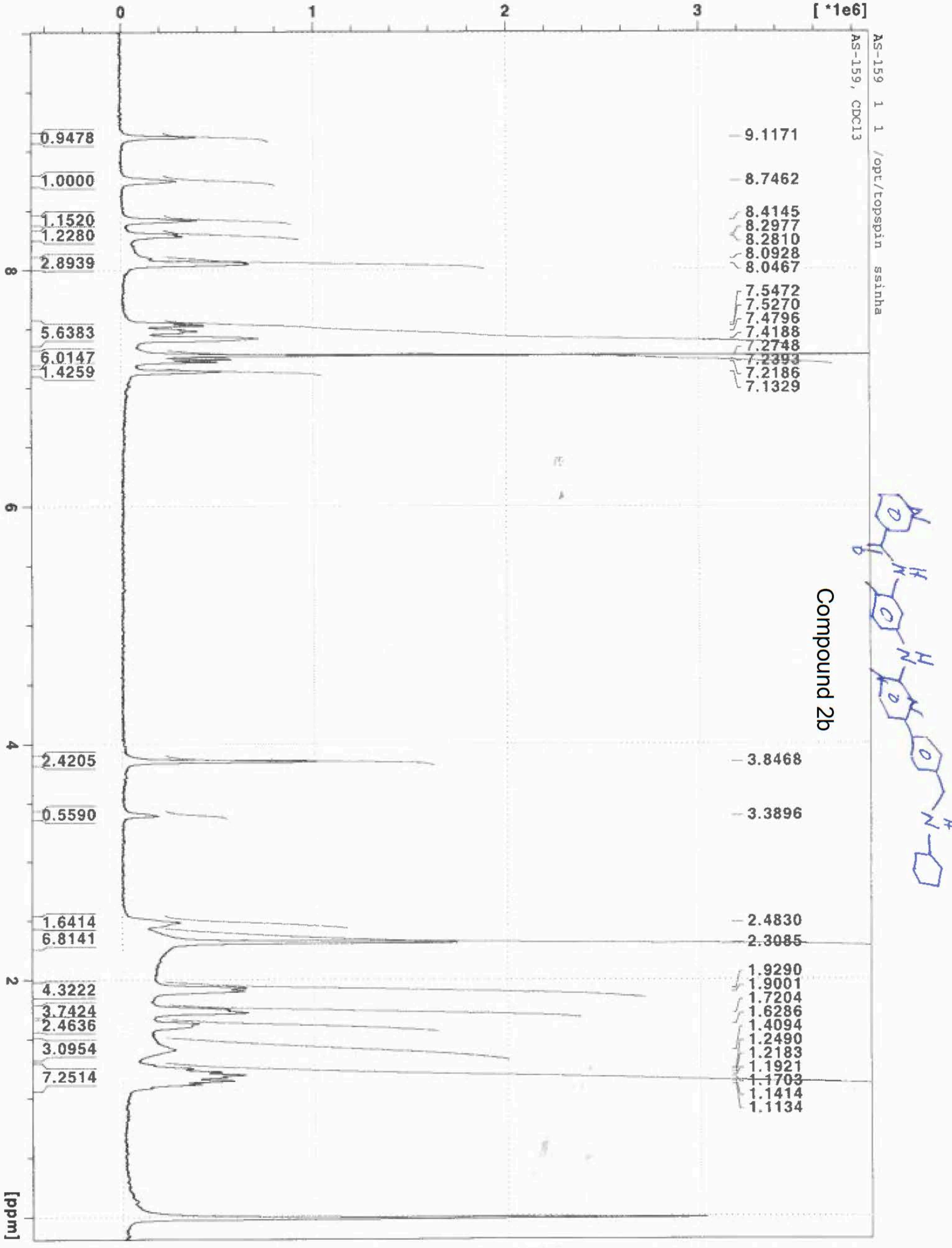

AS-159 1 1 /opt/topspin ssinha
AS-159, CDCl3
[*1e6]
[ppm]
9.1171
8.7462
8.4145
8.2977
8.2810
8.0928
8.0467
7.5472
7.5270
7.4796
7.4188
7.2748
7.2393
7.2186
7.1329
3.8468
3.3896
2.4830
2.3085
1.9290
1.9001
1.7204
1.6286
1.4094
1.2490
1.2183
1.1921
1.1703
1.1414
1.1134
0.9478
1.0000
1.1520
1.2280
2.8939
5.6383
6.0147
1.4259
2.4205
0.5590
1.6414
6.8141
4.3222
3.7424
2.4636
3.0954
7.2514
Compound 2b

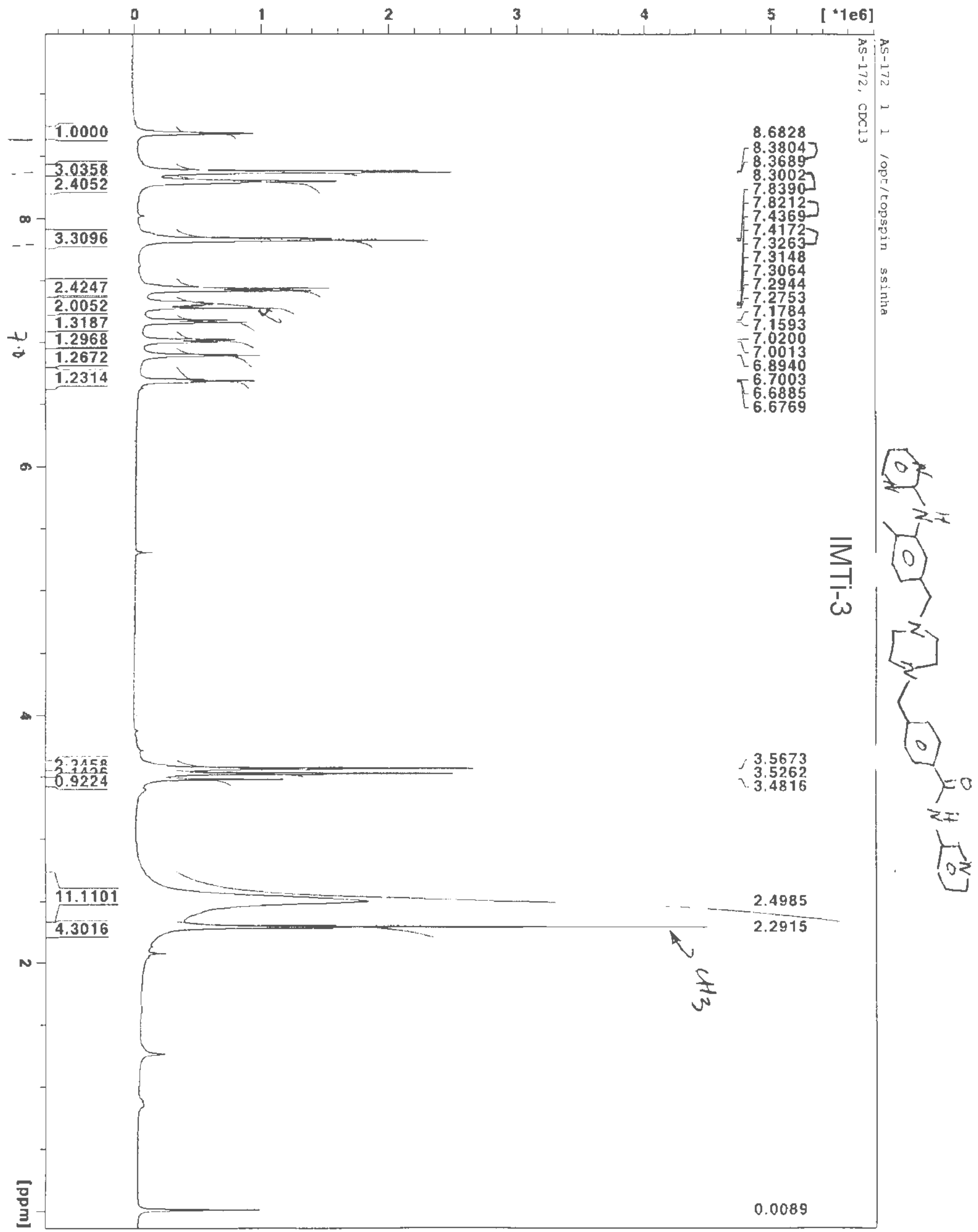
AS-172 1 1 /opt/topspin ssinha
AS-172, CDCl3
IMTi-3
[*1e6]
[ppm]
8.6828
8.3804
8.3689
8.3002
7.8390
7.8212
7.4369
7.4172
7.3263
7.3148
7.3064
7.2944
7.2753
7.1784
7.1593
7.0200
7.0013
6.8940
6.7003
6.6885
6.6769
3.5673
3.5262
3.4816
2.4985
2.2915
0.0089
1.0000
3.0358
2.4052
3.3096
2.4247
2.0052
1.3187
1.2968
1.2672
1.2314
0.9224
11.1101
4.3016
